# On compositions of special cases of Lipschitz continuous operators

**DOI:** 10.1186/s13663-021-00709-0

**Published:** 2021-12-20

**Authors:** Pontus Giselsson, Walaa M. Moursi

**Affiliations:** 1 Department of Automatic Control, Lund University, Lund, Sweden; 2 Department of Combinatorics and Optimization, University of Waterloo, Waterloo, Ontario N2L 3G1 Canada; 3 Mathematics Department, Faculty of Science, Mansoura University, Mansoura, 35516 Egypt

**Keywords:** 47H05, 90C25, 47H09, 49M27, 65K05, 65K10, Compositions of operators, Conically nonexpansive operators, Douglas–Rachford algorithm, Forward-backward algorithm, Hypoconvex function, Maximally monotone operator, Proximal operator, Resolvent

## Abstract

Many iterative optimization algorithms involve compositions of special cases of Lipschitz continuous operators, namely firmly nonexpansive, averaged, and nonexpansive operators. The structure and properties of the compositions are of particular importance in the proofs of convergence of such algorithms. In this paper, we systematically study the compositions of further special cases of Lipschitz continuous operators. Applications of our results include compositions of scaled conically nonexpansive mappings, as well as the Douglas–Rachford and forward–backward operators, when applied to solve certain structured monotone inclusion and optimization problems. Several examples illustrate and tighten our conclusions.

## Introduction

In this paper, we assume that

$$ \boxed{\text{$X$ is a real Hilbert space}} $$

with the inner product $\langle\cdot\mid\cdot\rangle$ and the induced norm $\Vert \cdot \Vert $. Let $L>0$ and let $T\colon X\to X$. Then *T* is *L*-*Lipschitz continuous* if $(\forall(x,y)\in X\times X)$ $\Vert Tx-Ty \Vert \le L \Vert x-y \Vert $, and *T* is *nonexpansive* if *T* is 1-Lipschitz continuous, i.e., $(\forall(x,y)\in X\times X)$ $\Vert Tx-Ty \Vert \le \Vert x-y \Vert $. In this paper, we study compositions of what we call (see Definition 3.1) identity-nonexpansive decompositions (I-N decompositions for short) of Lipschitz continuous operators. Let $(\alpha,\beta)\in{\mathbb{R}}^{2}$ and let $\operatorname{Id}\colon X\to X$ be the *identity operator* on *X*. A Lipschitz continuous operator *R* admits an $(\alpha,\beta)$-I-N decomposition if $R=\alpha{\mathrm{Id}}+\beta N$ for some nonexpansive operator $N\colon X\to X$. For instance, averaged,^1^ conically nonexpansive,^2^ and cocoercive^3^ operators are all Lipschitz continuous operators that admit special I-N decompositions.

We consider compositions of the form

1 $$ R=R_{m}\cdots R_{1}, $$

where $m\in\{2,3,\ldots\}$, $I=\{1,\ldots, m\}$, and $(R_{i})_{i\in I}$ is a family of Lipschitz continuous operators such that, for each $i\in I$, $R_{i}$ admits an $(\alpha_{i},\beta_{i})$-I-N decomposition. That is, $R_{i}=\alpha_{i}\operatorname{Id}+\beta_{i}N_{i}$ for all $i\in I$, where $\alpha_{i}$ and $\beta_{i}$ are real numbers, and $N_{i}\colon X\to X$ are nonexpansive for all $i\in I$. A straightforward (and naive) conclusion is that the composition is Lipschitz continuous with a constant $\Pi_{i\in I} ( \vert \alpha_{i} \vert + \vert \beta_{i} \vert )$. However, such a conclusion can be further refined when, for instance, each $R_{i}$ is an averaged operator. Indeed, in this case it is known that the composition is an averaged (and not just Lipschitz continuous) operator (see, e.g., [2, Proposition 4.46], [6, Lemma 2.2], and [21, Theorem 3]). In this paper, we provide a systematic study of the structure of *R* under additional assumptions on the decomposition parameters.

Our main result is stated in Theorem 3.4. We show that, for $m=2$, under a mild assumption on $(\alpha_{1},\alpha_{2},\beta_{1},\beta_{2})$ composition (1) is a scalar multiple of a conically nonexpansive operator. As a consequence of Theorem 3.4, we show in Theorem 4.2 that, under additional assumptions on the decomposition parameters, compositions of scaled conically nonexpansive mappings are scaled conically nonexpansive mappings, see also [1] for a relevant result.^4^ Special cases of Theorem 4.2 include, e.g., compositions of averaged operators [2, Proposition 4.46] and compositions of averaged and negatively averaged operators [12].

Of particular interest are compositions *R* that are averaged, conically nonexpansive, or contractive. Let $x_{0}\in X$. For an averaged (respectively contractive) operator *R*, the sequence $(R^{k}x_{0})_{k\in{\mathbb{N}}}$ converges weakly (respectively strongly) towards a fixed point of *R* (if one exists) [2, Theorem 5.14]. For conically nonexpansive operators, a simple averaging trick gives an averaged operator with the same fixed point set as the conically nonexpansive operator. Iterating the new averaged operator yields a sequence that converges weakly to a fixed point of the conically nonexpansive operator. These properties have been instrumental in proving convergence for the Douglas–Rachford algorithm and the forward–backward algorithm. In this paper, we apply our composition result Theorem 4.2 to prove convergence of these splitting methods in new settings.

The Douglas–Rachford and forward–backward methods traditionally solve monotone inclusion problems of the form

2 $$ \text{Find $x\in X$ such that $0\in Ax+Bx$}, $$

where $A\colon X\rightrightarrows X$ and $B\colon X\rightrightarrows X$ are maximally monotone, and, in the case of the forward–backward method, *A* is additionally assumed to be cocoercive. The Douglas–Rachford method iterates the Douglas–Rachford map $T=\frac{1}{2}({\mathrm{Id}}+R_{\gamma B}R_{\gamma A})$, where^5^$\gamma>0$ is a positive step-size. The Douglas–Rachford map is an averaged map of the composition of reflected resolvents. The forward–backward method iterates the forward–backward map $T=J_{\gamma B}({\mathrm{Id}}-\gamma A)$, where $\gamma>0$ is a positive step-size. The forward–backward map is a composition of a resolvent and a forward-step.

In this paper, we show that for Douglas–Rachford splitting we need not impose monotonicity on the individual operators, but only on the sum, provided the sum is strongly monotone. The reflected resolvents $R_{\gamma A}$ and $R_{\gamma B}$ are negatively conically nonexpansive, the composition is conically nonexpansive, and a sufficient averaging gives an averaged map that converges to a fixed point when iterated. Relevant work appears in [9, 16], and [17].

More striking, for the forward–backward method, we show that it is sufficient that the sum is monotone (not strongly monotone as for DR). More specifically, we show that identity can be shifted between the two operators, while still guaranteeing averagedness of the forward–backward map $T=J_{\gamma B}({\mathrm{Id}}-\gamma A)$. Indeed, the resolvent $J_{\gamma B}$ is cocoercive and the forward-step $({\mathrm{Id}}-\gamma A)$ is scaled averaged. This implies that the composition is averaged (given restrictions on the cocoercivity and averagedness parameters). Moreover, when the sum is strongly monotone, again with no assumptions on monotonicity of the individual operators, we show that the forward–backward map is contractive. We also prove tightness of our contraction factor.

We also provide, in Theorem 4.7, a generalization of Theorem 4.2 to the setting in (1) of compositions of more than two operators. We assume that all $R_{i}$ are scaled conically nonexpansive operators and provide conditions on the parameters that give a specific scaled conically nonexpansive representation of *R*. Our condition is symmetric in the individual operators and allows for one of them to be scaled conic, while the rest must be scaled averaged. This is in compliance with the $m=2$ case in Theorem 4.2.

Finally, in Sect. 8, we provide graphical 2D-representations of different operator classes that admit I-N decompositions such as Lipschitz continuous operators, averaged operators, and cocoercive operators. We also provide 2D-representations of compositions of two such operator classes. Illustrations of the firmly nonexpansive ($\frac {1}{2}$-averaged ) and nonexpansive operator classes have previously appeared in [10, 11], and illustrations of more operator classes that admit particular I-N decompositions and their compositions have appeared in [14, 24] and in early preprints of [15].

### Organization and notation

The remainder of this paper is organized as follows: Sect. 2 presents useful facts and auxiliary results that are used throughout the paper. In Sect. 3, we present the main abstract results of the paper. Section 4 presents the main composition results of Lipschitz continuous operators that admit I-N decompositions, under mild assumptions on the decomposition parameters, as well as illustrative and limiting examples. In Sect. 5 and Sect. 6, we present applications of our composition results to the Douglas–Rachford and forward–backward algorithms, respectively. In Sect. 7 we present applications of our results to optimization problems. Finally, in Sect. 8, we provide graphical representations of many different I-N decompositions and their compositions.

The notation we use is standard and follows, e.g., [2] or [23].

## Facts and auxiliary results

Let $\rho\in{\mathbb{R}}$. Let $A\colon X\to X$. Recall that *A* is *ρ*-*monotone* if $(\forall(x,u)\in\operatorname{gra}A)$ $(\forall(y,v)\in\operatorname{gra}A)$

3 $$ \langle x-y \mid u-v \rangle\ge\rho \Vert x-y \Vert ^{2} $$

and is maximally *ρ*-monotone if any proper extension of gra*A* will violate (3). In passing we point out that *A* is (maximally) monotone (respectively *ρ*-hypomonotone, *ρ*-strongly monotone) if $\rho=0$ (respectively $\rho<0$, $\rho>0$) see, e.g., [2, Chap. 20], [4, Definition 6.9.1], [7, Definition 2.2], and [23, Example 12.28].

### Fact 2.1

*Let* $A\colon X\rightrightarrows X$, *let* $B\colon X\rightrightarrows X$, *let* $\lambda\in{\mathbb{R}}\smallsetminus\{0\}$, *and suppose that* $\operatorname{zer}(A+B)= (A+B)^{-1}(0)\neq\varnothing$. *Suppose that* $J_{A}$ *and* $J_{B}$ *are single*-*valued and that* $\operatorname{dom}J_{A}=\operatorname{dom}J_{B}=X$. *Set*

4 $$ T=(1-\lambda)\operatorname{Id}+\lambda R_{B}R_{A}. $$

*Then* *T* *is single*-*valued*, $\operatorname{dom}T=X$, *and*

5 $$ \operatorname{zer}(A+B)=J_{A}(\operatorname{Fix}R_{B}R_{A})=J_{A}( \operatorname{Fix}T). $$

### Proof

See [9, Lemma 4.1]. □

### Proposition 2.2

*Let* $A\colon X\to X$, *let* $B\colon X\rightrightarrows X$, *and suppose that* $\operatorname{zer}(A+B)= (A+B)^{-1}(0)\neq\varnothing$. *Suppose that* $J_{B}$ *is single*-*valued and that* $\operatorname{dom}J_{B}=X$. *Set*

6 $$ T=J_{B}(\operatorname{Id}-A). $$

*Then* *T* *is single*-*valued*, $\operatorname{dom}T=X$, *and*

7 $$ \operatorname{zer}(A+B)= \operatorname{Fix}T. $$

### Proof

The proof is similar to the proof of [2, Proposition 26.1(iv)].^6^ Indeed, let $x\in X$. Then $x\in\operatorname{zer}(A+B)$ ⇔ $-Ax\in Bx$ ⇔ $(\operatorname{Id}-A)x\in(\operatorname{Id}+B)x$ ⇔ $x=J_{B}(\operatorname{Id}-A)x=Tx$. □

### Lemma 2.3

*Let* $\lambda\in{\mathbb{R}}$, *let* $R_{1}\colon X\to X$, *let* $R_{2}\colon X\to X$, *and set*

8 $$ R(\lambda)=(1-\lambda)\operatorname{Id}+\lambda R_{2}R_{1}. $$

*Let* $(x,y)\in X\times X$. *Then*

9 $$\begin{aligned} & \bigl\langle R(\lambda) x-R(\lambda) y \mid\bigl(\operatorname {Id}-R(\lambda)\bigr)x- \bigl( \operatorname {Id}-R(\lambda)\bigr)y \bigr\rangle \\ &\quad =(1-2\lambda)\bigl\langle x-y \mid\bigl(\operatorname {Id}-R(\lambda)\bigr)x-\bigl( \operatorname {Id}-R( \lambda)\bigr)y \bigr\rangle \\ &\qquad {} +\lambda^{2}\bigl\langle (\operatorname {Id}+R_{1})x-( \operatorname {Id}+R_{1})y \mid( \operatorname {Id}-R_{1})x-( \operatorname {Id}-R_{1})y \bigr\rangle \\ &\qquad {} +\lambda^{2}\bigl\langle (\operatorname {Id}+R_{2})R_{1}x-( \operatorname {Id}+R_{2})R_{1}y \mid(\operatorname {Id}-R_{2})R_{1}x-( \operatorname {Id}-R_{2})R_{1}y \bigr\rangle . \end{aligned}$$

### Proof

See Appendix A. □

### Proposition 2.4

*Let* $\alpha\in{\mathbb{R}}$, *let* $\beta\in{\mathbb{R}}$, *let* $N\colon X\to X$, *and set* $T=\alpha{\mathrm{Id}}+\beta N$. *Let* $(x,y)\in X\times X$. *Then the following hold*:

10a $$\begin{aligned} &\beta^{2}\bigl( \Vert x-y \Vert ^{2}- \Vert Nx-Ny \Vert ^{2}\bigr) \\ &\quad = \bigl(\beta^{2}-\alpha^{2}\bigr) \Vert x-y \Vert ^{2}- \Vert Tx-Ty \Vert ^{2}+2\alpha\langle x-y \mid Tx-Ty \rangle \end{aligned}$$

10b $$\begin{aligned} &\quad = \bigl(\beta^{2}-\alpha^{2}\bigr) \Vert x-y \Vert ^{2}-(1-2\alpha) \Vert Tx-Ty \Vert ^{2}+2\alpha\bigl\langle Tx-Ty \mid(\operatorname {Id}-T)x-( \operatorname {Id}-T)y \bigr\rangle \end{aligned}$$

10c $$\begin{aligned} &\quad = \bigl(\beta^{2}-\alpha(\alpha-1)\bigr) \Vert x-y \Vert ^{2} - \bigl((1- \alpha) \Vert Tx-Ty \Vert ^{2}+ \alpha \bigl\Vert (\operatorname {Id}-T)x-( \operatorname {Id}-T)y \bigr\Vert ^{2} \bigr). \end{aligned}$$

### Proof

Indeed, we have

11a $$\begin{aligned} & \beta^{2}\bigl( \Vert x-y \Vert ^{2}- \Vert Nx-Ny \Vert ^{2} \bigr) \\ &\quad =\beta^{2} \Vert x-y \Vert ^{2}- \bigl\Vert (Tx- \alpha x)-(Ty-\alpha y) \bigr\Vert ^{2} \end{aligned}$$

11b $$\begin{aligned} &\quad =\beta^{2} \Vert x-y \Vert ^{2}-\bigl( \Vert Tx-Ty \Vert ^{2}+\alpha^{2} \Vert x-y \Vert ^{2}-2 \alpha\langle Tx-Ty \mid x-y \rangle\bigr) \end{aligned}$$

11c $$\begin{aligned} &\quad =\bigl({\beta^{2}}-{\alpha^{2}}\bigr) \Vert x-y \Vert ^{2}- \bigl( \Vert Tx-Ty \Vert ^{2}-2\alpha \langle Tx-Ty \mid x-y \rangle \bigr) \end{aligned}$$

11d $$\begin{aligned} &\quad =\bigl({\beta^{2}}-{\alpha^{2}}+\alpha\bigr) \Vert x-y \Vert ^{2}- \bigl((1-\alpha) \Vert Tx-Ty \Vert ^{2} \\ &\qquad {} +\alpha \Vert Tx-Ty \Vert ^{2} -2\alpha\langle Tx-Ty \mid x-y \rangle+ \alpha \Vert x-y \Vert ^{2} \bigr) \end{aligned}$$

11e $$\begin{aligned} &\quad = \bigl(\beta^{2}-\alpha(\alpha-1)\bigr) \Vert x-y \Vert ^{2} - \bigl((1- \alpha) \Vert Tx-Ty \Vert ^{2}+ \alpha \bigl\Vert (\operatorname {Id}-T)x-( \operatorname {Id}-T)y \bigr\Vert ^{2} \bigr). \end{aligned}$$

This proves (10a) and (10c) in view of (11c) and (11e). Finally, note that $(\beta^{2}-\alpha^{2}) \Vert x-y \Vert ^{2}- \Vert Tx-Ty \Vert ^{2}+2\alpha\langle x-y \mid Tx-Ty \rangle=(\beta^{2}- \alpha^{2}) \Vert x-y \Vert ^{2}-(1-2\alpha) \Vert Tx-Ty \Vert ^{2} -2\alpha \Vert Tx-Ty \Vert ^{2}+2\alpha\langle x-y \mid Tx-Ty \rangle= (\beta^{2}-\alpha^{2}) \Vert x-y \Vert ^{2}-(1-2 \alpha) \Vert Tx-Ty \Vert ^{2}+2\alpha\langle Tx-Ty \mid( \operatorname {Id}-T)x-(\operatorname {Id}-T)y \rangle$. This proves (10b). □

### Proposition 2.5

*Let* $\alpha\in{\mathbb{R}}$, *let* $\beta\in{\mathbb{R}}$, *let* $N\colon X\to X$, *and set* $T=\alpha{\mathrm{Id}}+\beta N$. *Let* $(x,y)\in X\times X$. *Then the following are equivalent*:

(i) *N* *is nonexpansive*.
(ii) $\|Tx-Ty\|^{2}-2\alpha\langle x-y \mid Tx-Ty \rangle\leq(\beta^{2}- \alpha^{2})\|x-y\|^{2}$.
(iii) $(1-2\alpha)\|Tx-Ty\|^{2}-2\alpha\langle Tx-Ty \mid({\mathrm{Id}}-T)x-( {\mathrm{Id}}-T)y \rangle\leq(\beta^{2}-\alpha^{2})\|x-y\|^{2}$.
(iv) $(2\alpha-1)\|({\mathrm{Id}}-T)x-({\mathrm{Id}}-T)y\| ^{2}-2(1-\alpha) \langle Tx-Ty \mid({\mathrm{Id}}-T)x-({\mathrm{Id}}-T)y \rangle\leq (\beta^{2}-(1- \alpha)^{2})\|x-y\|^{2}$.
(v) $(1-\alpha)\|Tx-Ty\|^{2} +\alpha\|({\mathrm{Id}}-T)x-({\mathrm {Id}}-T)y\|^{2} \leq(\beta^{2}-\alpha(\alpha-1))\|x-y\|^{2} $.

### Proof

(i)⇔(ii)⇔(iii)⇔(v): This is a direct consequence of Proposition 2.4. (i)⇔(iv): Applying (10b) with $(T,\alpha,\beta)$ replaced by $(\operatorname{Id}-T, 1-\alpha,-\beta)$ yields $\beta^{2}( \Vert x-y \Vert ^{2}- \Vert Nx-Ny \Vert ^{2}) = (\beta^{2}-(1-\alpha)^{2})\|x-y\|^{2} -(2\alpha-1)\|( \operatorname{Id}-T)x-(\operatorname{Id}-T)y\|^{2}+2(1-\alpha) \langle Tx-Ty \mid({\mathrm{Id}}-T)x-({\mathrm{Id}}-T)y \rangle$. The proof is complete. □

### Proposition 2.6

*Let* $\alpha\in{\mathbb{R}}$, *let* $N\colon X\to X$, *and set* $T=(1-\alpha)\operatorname{Id}+\alpha N$. *Let* $(x,y)\in X\times X$. *Then the following are equivalent*:

(i) *N* *is nonexpansive*.
(ii) $\|Tx-Ty\|^{2}-2(1-\alpha)\langle x-y \mid Tx-Ty \rangle\leq(2 \alpha-1)\|x-y\|^{2}$.
(iii) $(2\alpha-1)\|Tx-Ty\|^{2}-2(1-\alpha)\langle Tx-Ty \mid({\mathrm{Id}}-T)x-( {\mathrm{Id}}-T)y \rangle\leq(2\alpha-1)\|x-y\|^{2}$.
(iv) $(1-2\alpha)\|({\mathrm{Id}}-T)x-({\mathrm{Id}}-T)y\|^{2}\leq 2\alpha \langle Tx-Ty \mid({\mathrm{Id}}-T)x-({\mathrm{Id}}-T)y \rangle$.
(v) $(1-\alpha)\|({\mathrm{Id}}-T)x-({\mathrm{Id}}-T)y\|^{2} \leq \alpha\|x-y\|^{2} -\alpha\|Tx-Ty\|^{2}$.

### Proof

Apply Proposition 2.5 with $(\alpha,\beta)$ replaced by $(1-\alpha,\alpha)$. □

### Lemma 2.7

*Let* $\lambda<1$. *Then*

12 $$ \Vert x \Vert ^{2}-\lambda \Vert y \Vert ^{2} \geq-\frac{\lambda}{1-\lambda} \Vert x+y \Vert ^{2}. $$

### Proof

Let $\delta>0$. By Young’s inequality, $\|x+y\|^{2}=\|x\|^{2}+2\langle x,y\rangle+\|y\|^{2}\geq(1-\delta) \|x\|^{2}+(1-\delta^{-1})\|y\|^{2} $. Equivalently, $\|x+y\|^{2}-(1-\delta)\|x\|^{2}\geq(1-\delta^{-1})\|y\|^{2}$. Now, replace $(x,y,\delta)$ by $(-y,x+y,1-\lambda)$. □

### Proposition 2.8

*Let* $\alpha\in\, ]0,1 [$, *let* $\beta>0$, *and let* $T\colon X\to X$. *Then* *T* *is* *α*-*averaged if and only if* $T=(1-\beta)\operatorname{Id}+\beta M$ *and* *M* *is* $\frac{\alpha}{\beta}$-*conically nonexpansive*.

### Proof

Indeed, *T* is *α*-averaged if and only if there exists a nonexpansive mapping $N\colon X\to X$ such that $T=(1-\alpha){\mathrm{Id}}+\alpha N$. Equivalently,

$$ T=(1-\alpha){\mathrm{Id}}+\alpha N=(1-\beta)\operatorname{Id}+\beta \biggl( \biggl(1-\frac{\alpha}{\beta}\biggr)\operatorname{Id}+ \frac{\alpha}{\beta}N \biggr), $$

and the conclusion follows by setting $M= (1-\frac{\alpha}{\beta} )\operatorname{Id}+ \frac{\alpha}{\beta}N$. □

The following three lemmas can be directly verified, hence we omit the proof.

### Lemma 2.9

*Let* $\alpha>0$, *and let* $T\colon X\to X$. *Then* *T* *is* *α*-*conically nonexpansive* ⇔ $\operatorname{Id}-T$ *is* $\frac{1}{2\alpha} $-*cocoercive* ⇒ $\operatorname{Id}-T$ *is maximally monotone*.

### Lemma 2.10

*Let* $\beta>0$, *let* $\mu\in{\mathbb{R}}$, *and let* $A\colon X\to X$. *Suppose that* *A* *is maximally* *μ*-*monotone and* $\frac{1}{\beta} $-*cocoercive*. *Then* $\mu\le\frac{1}{\beta} $.

### Lemma 2.11

*Let* $\beta>0$, *let* $T\colon X\to X$, *and let* $\overline{\beta}\ge\beta$. *Suppose that* *T* *is* $\frac{1}{\beta} $-*cocoercive*. *Then* *T* *is* $\frac{1}{\overline{\beta}} $-*cocoercive*.

### Lemma 2.12

*Let* $\beta>0$, *and let* $A\colon X\to X$. *Suppose that* *A* *is* *β*-*Lipschitz continuous*. *Then the following hold*:

(i) *A* *is maximally* $(-{\beta})$-*monotone*.
(ii) $A+{\beta}\operatorname{Id}$ *is* $\frac{1}{2\beta}$-*cocoercive*.

### Proof

See Appendix B. □

### Lemma 2.13

*Let* $\beta>\delta>0$, *let* $T_{1}\colon X\to X$, *and let* $T_{2}\colon X\to X$. *Suppose that* $T_{1}$ (*respectively* $T_{2}$) *is* $\frac{1}{\beta} $-*cocoercive* (*respectively* $\frac{1}{\delta}$-*cocoercive*). *Then* $T_{1}-T_{2}$ *is* *β*-*Lipschitz continuous*.

### Proof

See Appendix C. □

As a corollary, we obtain the following result which was stated in [27, page 4].

### Corollary 2.14

*Let* $f_{1}\colon X\to{\mathbb{R}}$, $f_{2}\colon X\to{\mathbb{R}}$ *be Frechét differentiable convex functions*, *and let* $\beta>\delta>0$. *Suppose that* $\mathop{\nabla{f} } _{1}$ (*respectively* $\mathop{\nabla{f} } _{2}$) *is* *β*-*Lipschitz continuous* (*respectively* *δ*-*Lipschitz continuous*). *Then the following hold*:

(i) $\mathop{\nabla{f} } _{1}-\mathop{\nabla{f} } _{2}$ *is* *β*-*Lipschitz continuous*.
(ii) *Suppose that* $f_{1}-f_{2}$ *is convex*. *Then* $\mathop{\nabla{f} } _{1}-\mathop{\nabla{f} } _{2}$ *is* $\frac{1}{\beta}$-*cocoercive*.

### Proof

See Appendix D. □

### Lemma 2.15

*Let* $\alpha\in\, ]0,1 [$, *let* $\delta\in\, ]0,1 ]$, *and let* $T\colon X\to X$. *Suppose that* *T* *is* *α*-*averaged*. *Then the following hold*:

(i) *δT* *is* $(1-\delta(1-\alpha))$-*averaged*.
(ii) *Suppose that* $\delta\in\, ]0,1 [$. *Then* *δT* *is a Banach contraction with constant* *δ*.

### Proof

See Appendix E. □

Let *A* be maximally *ρ*-*monotone*, where $\rho>-1$. Then (see [9, Proposition 3.4] and [3, Corollary 2.11 and Proposition 2.12]) we have

13 $$ \text{$J_{A}$ is single-valued and $\operatorname{dom}J_{A}=X$.} $$

The following result involves resolvents and reflected resolvents of *ρ*-monotone operators.

### Proposition 2.16

*Let* *A* *be* *ρ*-*monotone*, *where* $\rho>-1$. *Then the following hold*:

(i) $J_{A}$ *is* $(1+\rho)$- *cocoercive*, *in which case* $J_{A}$ *is Lipschitz continuous with constant* $\frac{1}{1+\rho}$.
(ii) $-R_{A}$ *is* $\frac{1}{1+\rho}$-*conically nonexpansive*.
(iii) *Suppose that* $\rho\le0$. *Then* $R_{A}$ *is Lipschitz continuous with constant* $\frac{1- \rho}{1+\rho} $.

### Proof

(i): See [9, Lemma 3.3(ii)]. Alternatively, it follows from [3, Corollary 3.8(ii)] that $\operatorname{Id}-T$ is $\frac{1}{2(1+\rho)}$-averaged. Now apply Lemma 2.9 with *T* replaced by $\operatorname{Id}-J_{A}$. (ii): It follows from (i) that there exists a nonexpansive operator $N\colon X\to X $ such that $J_{A}=\frac{1}{2(1+\rho)}(\operatorname{Id}+N)$. Now, $-R_{A}=\operatorname{Id}-2J_{A}=\operatorname{Id}- \frac{1}{1+\rho}(\operatorname{Id}+N) = (1-\frac{1}{1+\rho} )\operatorname{Id}+\frac{1}{1+\rho}N$. (iii): Indeed, let $(x,y)\in X\times X$ and let *N* be as defined above. We have

14a $$\begin{aligned} \Vert R_{A}x-R_{A}y \Vert &= \biggl\Vert - \frac{\rho}{1+\rho}(x-y)- \frac{1}{1+\rho}(Nx-Ny) \biggr\Vert \leq- \frac{\rho}{1+\rho} \Vert x-y \Vert +\frac{1}{1+\rho} \Vert Nx-Ny \Vert \end{aligned}$$

14b $$\begin{aligned} &\leq\frac{1-\rho}{1+\rho} \Vert x-y \Vert . \end{aligned}$$

The proof is complete. □

## Compositions

### Definition 3.1

($(\alpha,\beta)$-I-N decomposition)

Let $R\colon X\to X$ be Lipschitz continuous, and let^7^$(\alpha,\beta)\in{\mathbb{R}}\times{\mathbb{R}}_{+}$. We say that *R* admits an $(\alpha,\beta)$-identity-nonexpansive (I-N) decomposition^8^ if there exists a nonexpansive operator $N\colon X\to X$ such that $R=\alpha\operatorname{Id}+\beta N$.

Throughout the rest of this paper, we assume that

$$ \boxed{\text{$R_{1}\colon X\to X$ and $R_{2}\colon X\to X$ are Lipschitz continuous operators.}} $$

### Proposition 3.2

*Let* $\alpha_{1}\in\, ]{-}\infty,1 [$, *let* $\alpha_{2}\in\, ]{-}\infty,1 [$, *let* $\beta_{1}\in{\mathbb{R}}_{+}$, *let* $\beta_{2}\in{\mathbb {R}}_{+}$, *and suppose that* $\alpha_{2}(\alpha_{2}-1)\le\beta_{2}^{2}$. *Set*

15a $$\begin{aligned}& \delta_{1} = \frac{\alpha_{1}}{1-\alpha_{1}} \biggl(1- \frac{(1-\alpha_{2})^{2}-\beta_{2}^{2}}{1-\alpha_{2}} \biggr), \end{aligned}$$

15b $$\begin{aligned}& \delta_{2} =\frac{\alpha_{2}}{1-\alpha_{2}}, \end{aligned}$$

15c $$\begin{aligned}& \delta_{3} =1- \biggl( \frac{(1-\alpha_{1})^{2}-\beta_{1}^{2}}{1-\alpha_{1}} \biggl(1- \frac{(1-\alpha_{2})^{2}-\beta_{2}^{2}}{1-\alpha_{2}} \biggr) + \frac{(1-\alpha_{2})^{2}-\beta_{2}^{2}}{(1-\alpha_{2})} \biggr). \end{aligned}$$

*Suppose that* $R_{1}$ *admits an* $(\alpha_{1},\beta_{1})$-*I*-*N decomposition and that* $R_{2}$ *admits an* $(\alpha_{2},\beta_{2})$-*I*-*N decomposition*. *Then* $(\forall(x,y)\in X \times X)$ *we have*

16 $$\begin{aligned}& \Vert R_{2}R_{1}x-R_{2}R_{1}y \Vert ^{2} +\delta_{1} \bigl\Vert ( \operatorname {Id}-R_{1})x-(\operatorname {Id}-R_{1})y \bigr\Vert ^{2} \\& \quad {}+\delta_{2} \bigl\Vert (\operatorname {Id}-R_{2})R_{1}x-( \operatorname {Id}-R_{2})R_{1}y \bigr\Vert ^{2} \leq \delta_{3} \Vert x-y \Vert ^{2}. \end{aligned}$$

### Proof

Set $T_{i}=\frac{1}{2}({\mathrm{Id}}+R_{i})=\frac{1+\alpha_{i}}{2} \operatorname{Id}+\frac{\beta_{i}}{2} N_{i}$, and observe that by Proposition 2.5 applied with $(T, \alpha,\beta)$ replaced by $(T_{i},\frac{1+\alpha_{i}}{2},\frac{\beta_{i}}{2} )$, $i\in\{1,2\}$, we have $(\forall(x,y)\in X\times X)$

17 $$\begin{aligned}& \bigl\langle T_{i}x-T_{i}y \mid( \operatorname {Id}-T_{i})x-(\operatorname {Id}-T_{i})y \bigr\rangle \\& \quad \ge \frac{\alpha_{i}}{1-\alpha_{i}} \bigl\Vert (\operatorname {Id}-T_{i})x-( \operatorname {Id}-T_{i})y \bigr\Vert ^{2} + \frac{(1-\alpha_{i})^{2}-\beta_{i}^{2}}{4(1-\alpha_{i})} \Vert x-y \Vert ^{2}. \end{aligned}$$

Equivalently,

18 $$ \begin{aligned}[b] &\bigl\langle (\operatorname {Id}+R_{i})x-( \operatorname {Id}+R_{i})y \mid( \operatorname {Id}-R_{i})x-( \operatorname {Id}-R_{i})y \bigr\rangle \\ &\quad \ge\frac{\alpha_{i}}{1-\alpha_{i}} \bigl\Vert (\operatorname {Id}-R_{i})x-( \operatorname {Id}-R_{i})y \bigr\Vert ^{2} + \frac{(1-\alpha_{i})^{2}-\beta_{i}^{2}}{1-\alpha_{i}} \Vert x-y \Vert ^{2}. \end{aligned} $$

Observe also that, because $\alpha_{2}<1$, we have

19 $$ \alpha_{2}(\alpha_{2}-1)\le \beta_{2}^{2} \quad \Leftrightarrow\quad 1- \frac{(1-\alpha_{2})^{2}-\beta_{2}^{2}}{1-\alpha_{2}}\ge0. $$

It follows from (18), applied with $i=2$ and $(x,y)$ replaced by $(R_{1}x,R_{1}y)$ in (20c) and by $i=1$ in (20f), in view of (19) that

20a $$\begin{aligned}& \Vert x-y \Vert ^{2}- \Vert R_{2}R_{1}x-R_{2}R_{1}y \Vert ^{2} \\& \quad = \Vert x-y \Vert ^{2}- \Vert R_{1}x-R_{1}y \Vert ^{2}+ \Vert R_{1}x-R_{1}y \Vert ^{2}- \Vert R_{2}R_{1}x-R_{2}R_{1}y \Vert ^{2} \end{aligned}$$

20b $$\begin{aligned}& \quad =\bigl\langle (\operatorname {Id}+R_{1})x-(\operatorname {Id}+R_{1})y \mid({ \mathrm{Id}}-R_{1})x-( {\mathrm{Id}}-R_{1})y \bigr\rangle \\& \qquad {} +\bigl\langle (\operatorname {Id}+R_{2})R_{1}x-( \operatorname {Id}+R_{2})R_{1}y \mid( {\mathrm{Id}}-R_{2})R_{1}x-({ \mathrm{Id}}-R_{2})R_{1}y \bigr\rangle \end{aligned}$$

20c $$\begin{aligned}& \quad \ge\bigl\langle (\operatorname {Id}+R_{1})x-(\operatorname {Id}+R_{1})y \mid({\mathrm{Id}}-R_{1})x-( {\mathrm{Id}}-R_{1})y \bigr\rangle \\& \qquad {}+ \frac{\alpha_{2}}{1-\alpha _{2}} \bigl\Vert ( \operatorname {Id}-R_{2})R_{1}x-( \operatorname {Id}-R_{2})R_{1}y \bigr\Vert ^{2} +\frac{(1-\alpha_{2})^{2}-\beta_{2}^{2}}{1-\alpha_{2}} \Vert R_{1}x-R_{1}y \Vert ^{2} \end{aligned}$$

20d $$\begin{aligned}& \quad =\bigl\langle (\operatorname {Id}+R_{1})x-(\operatorname {Id}+R_{1})y \mid({ \mathrm{Id}}-R_{1})x-( {\mathrm{Id}}-R_{1})y \bigr\rangle \\& \qquad {}+ \frac{\alpha_{2}}{1-\alpha _{2}} \bigl\Vert ( \operatorname {Id}-R_{2})R_{1}x-( \operatorname {Id}-R_{2})R_{1}y \bigr\Vert ^{2} \\& \qquad {} +\frac{(1-\alpha_{2})^{2}-\beta_{2}^{2}}{1-\alpha_{2}} \bigl( \Vert x-y \Vert ^{2}-\bigl\langle (\operatorname {Id}+R_{1})x-(\operatorname {Id}+R_{1})y \mid({ \mathrm{Id}}-R_{1})x-({\mathrm{Id}}-R_{1})y \bigr\rangle \bigr) \end{aligned}$$

20e $$\begin{aligned}& \quad = \biggl(1-\frac{(1-\alpha_{2})^{2}-\beta_{2}^{2}}{1-\alpha_{2}} \biggr) \bigl\langle (\operatorname {Id}+R_{1})x-( \operatorname {Id}+R_{1})y \mid({\mathrm {Id}}-R_{1})x-( { \mathrm{Id}}-R_{1})y \bigr\rangle \\& \qquad {} +\frac{\alpha_{2}}{1-\alpha_{2}} \bigl\Vert (\operatorname {Id}-R_{2})R_{1}x-( \operatorname {Id}-R_{2})R_{1}y \bigr\Vert ^{2} + \frac{(1-\alpha_{2})^{2}-\beta_{2}^{2}}{1-\alpha_{2}} \Vert x-y \Vert ^{2} \end{aligned}$$

20f $$\begin{aligned}& \quad \ge \biggl(1-\frac{(1-\alpha_{2})^{2}-\beta_{2}^{2}}{1-\alpha_{2}} \biggr) \biggl( \frac{\alpha_{1}}{1-\alpha_{1}} \bigl\Vert (\operatorname {Id}-R_{1})x-( \operatorname {Id}-R_{1})y \bigr\Vert ^{2} + \frac{(1-\alpha_{1})^{2}-\beta_{1}^{2}}{1-\alpha_{1}} \Vert x-y \Vert ^{2} \biggr) \\& \qquad {} + \frac{\alpha_{2}}{1-\alpha_{2}} \bigl\Vert (\operatorname {Id}-R_{2})R_{1}x-( \operatorname {Id}-R_{2})R_{1}y \bigr\Vert ^{2} + \frac{(1-\alpha_{2})^{2}-\beta_{2}^{2}}{1-\alpha_{2}} \Vert x-y \Vert ^{2} \end{aligned}$$

20g $$\begin{aligned}& \quad = \frac{\alpha_{1}}{1-\alpha_{1}} \biggl(1- \frac{(1-\alpha_{2})^{2}-\beta_{2}^{2}}{1-\alpha_{2}} \biggr) \bigl\Vert ( \operatorname {Id}-R_{1})x-(\operatorname {Id}-R_{1})y \bigr\Vert ^{2} \\& \qquad {}+ \frac{\alpha_{2}}{1-\alpha_{2}} \bigl\Vert (\operatorname {Id}-R_{2})R_{1}x-( \operatorname {Id}-R_{2})R_{1}y \bigr\Vert ^{2} \\& \qquad {} + \biggl( \frac{(1-\alpha_{1})^{2}-\beta_{1}^{2}}{1-\alpha_{1}} \biggl(1- \frac{(1-\alpha_{2})^{2}-\beta_{2}^{2}}{1-\alpha_{2}} \biggr) + \frac{(1-\alpha_{2})^{2}-\beta_{2}^{2}}{1-\alpha_{2}} \biggr) \Vert x-y \Vert ^{2}. \end{aligned}$$

Rearranging yields the desired result. □

### Theorem 3.3

*Let* $\alpha_{1}\in\, ]{-}\infty,1 [$, *let* $\alpha_{2}\in\, ]{-}\infty,1 [$, *let* $\beta_{1}\in{\mathbb{R}}_{+}$, *let* $\beta_{2}\in{\mathbb {R}}_{+}$, *and suppose that* $\alpha_{2}(\alpha_{2}-1)\le\beta_{2}^{2}$. *Let* $\delta_{1}$, $\delta_{2}$, *and* $\delta_{3}$ *be defined as in* (15a)*–*(15c). *Set*

21 $$ \delta_{4} = \frac{\delta_{1}\delta_{2}}{\delta_{1}+\delta_{2}}, $$

*and suppose that* $\delta_{1}+\delta_{2}>0$, *that* $\delta_{3}-\delta_{4}+\delta_{3}\delta_{4}\ge0$, *and that* $\delta_{4}>-1$. *Suppose that* $R_{1}$ *admits an* $(\alpha_{1},\beta_{1})$-*I*-*N decomposition*, *and that* $R_{2}$ *admits an* $(\alpha_{2},\beta_{2})$-*I*-*N decomposition*. *Then* $R_{2}R_{1}$ *admits an* $(\alpha,\beta)$-*I*-*N decomposition*, *where*

22 $$ \alpha=\frac{\delta_{4}}{1+\delta_{4}},\qquad \beta= \frac{\sqrt{\delta_{3}-\delta_{4}+\delta_{3}\delta_{4}}}{1+\delta_{4}}. $$

### Proof

Let $\underline{\delta}:=\min(\delta_{1},\delta_{2})$, let $\bar{\delta}:=\max(\delta_{1},\delta_{2})$, and let $\lambda:=-\underline{\delta}/\bar{\delta}$ (i.e., $\lambda=-\delta_{1}/\delta_{2}$ if $\delta_{1}\leq\delta_{2}$, and $\lambda=-\delta_{2}/\delta_{1}$ if $\delta_{1}\geq\delta_{2}$). Then Proposition 3.2 and Lemma 2.7 imply that

23a $$\begin{aligned} & \delta_{3} \Vert x-y \Vert ^{2}- \Vert R_{2}R_{1}x-R_{2}R_{1}y \Vert ^{2} \\ &\quad \geq\delta_{1} \bigl\Vert (\operatorname {Id}-R_{1})x-( \operatorname {Id}-R_{1})y \bigr\Vert ^{2}+\delta_{2} \bigl\Vert (\operatorname {Id}-R_{2})R_{1}x-(\operatorname {Id}-R_{2})R_{1}y \bigr\Vert ^{2} \end{aligned}$$

23b $$\begin{aligned} &\quad =\bar{\delta}(\frac{\delta_{1}}{\bar{\delta}} \bigl\Vert (\operatorname {Id}-R_{1})x-( \operatorname {Id}-R_{1})y \bigr\Vert ^{2} +\frac{\delta_{2}}{\bar{\delta}} \bigl\Vert (\operatorname {Id}-R_{2})R_{1}x-( \operatorname {Id}-R_{2})R_{1}y \bigr\Vert ^{2} \end{aligned}$$

23c $$\begin{aligned} &\quad \geq\bar{\delta}\biggl(-\frac{\lambda}{1-\lambda} \bigl\Vert ( \operatorname {Id}-R_{1})x-( \operatorname {Id}-R_{1})y+(\operatorname {Id}-R_{2})R_{1}x-( \operatorname {Id}-R_{2})R_{1}y \bigr\Vert ^{2}\biggr) \end{aligned}$$

23d $$\begin{aligned} &\quad =-\frac{\lambda\bar{\delta}}{1-\lambda}\bigl\| ({\mathrm {Id}}-R_{2}R_{1})x-( {\mathrm{Id}}-R_{2}R_{1})y \bigr\| ^{2} \end{aligned}$$

23e $$\begin{aligned} &\quad = \frac{\underline{\delta}\bar{\delta}}{\bar{\delta}+\underline {\delta}} \bigl\| ({\mathrm{Id}}-R_{2}R_{1})x-({ \mathrm{Id}}-R_{2}R_{1})y \bigr\| ^{2} \end{aligned}$$

23f $$\begin{aligned} &\quad =\delta_{4}\bigl\| ({\mathrm{Id}}-R_{2}R_{1})x-({ \mathrm{Id}}-R_{2}R_{1})y \bigr\| ^{2}. \end{aligned}$$

Comparing (23a)–(23f) to Proposition 2.5 applied with *T* replaced by $R_{2}R_{1}$, we learn that there exist a nonexpansive operator $N\colon X\to X$ and $(\alpha,\beta)\in{\mathbb{R}}^{2}$ such that $R_{2}R_{1}=\alpha\operatorname{Id}+\beta N$, where $\delta_{3}=\frac{\beta^{2}+\alpha(1-\alpha)}{1-\alpha}$ and $\delta_{4}=\frac{\alpha}{1-\alpha}$. Equivalently, $\alpha=\frac{\delta_{4}}{1+\delta_{4}}$, hence $\beta= \frac{\sqrt{\delta_{3}-\delta_{4}+\delta_{3}\delta_{4}}}{1+\delta_{4}}$, as claimed. □

### Theorem 3.4

*Let* $\alpha_{1}\in{\mathbb{R}}$, *let* $\alpha_{2}\in{\mathbb{R}}$, *let* $\beta_{1}>0$, *let* $\beta_{2}>0$, *suppose that* ${\alpha_{1}}+\beta_{1}>0$, *that* ${\alpha_{2}}+\beta_{2}>0$, *and that either* $\frac{\beta_{1}\beta_{2}}{(\alpha_{1}+\beta_{1})(\alpha_{2}+\beta _{2})}<1$ *or* $\max \{\frac{\beta_{1}}{\alpha_{1}+\beta_{1}}, \frac{\beta_{2}}{\alpha_{2}+\beta_{2}} \}=1$. *Set*

24a $$\begin{aligned}& \kappa=(\alpha_{1}+\beta_{1}) (\alpha_{2}+ \beta_{2}), \end{aligned}$$

24b $$\begin{aligned}& \theta= \textstyle\begin{cases} \frac{\beta_{1}{\alpha_{2}}+\beta_{2}{\alpha_{1}}}{ {\alpha_{1}}{\alpha_{2}}+{\alpha_{1}}\beta_{2}+{\alpha_{2}}\beta_{1}}, & \frac{\beta_{1}\beta_{2}}{({\alpha_{1}}+\beta_{1})({\alpha _{2}}+\beta_{2})}< 1; \\ 1, & \max \{\frac{\beta_{1}}{{\alpha_{1}}+\beta_{1}}, \frac{\beta_{2}}{{\alpha_{2}}+\beta_{2}} \}=1. \end{cases}\displaystyle \end{aligned}$$

*Suppose that* $R_{1}$ *admits an* $(\alpha_{1},\beta_{1})$-*I*-*N decomposition*, *and that* $R_{2}$ *admits an* $(\alpha_{2},\beta_{2})$-*I*-*N decomposition*. *Then* $\theta\in\, ]0,+\infty [$ *and* $R_{2}R_{1}$ *admits a* $(\kappa(1-\theta),\kappa\theta)$-*I*-*N decomposition*, *i*.*e*., $R_{2}R_{1}$ *is* *κ*-*scaled* *θ*-*conically nonexpansive*. *That is*, *there exists a nonexpansive operator* $N\colon X\to X$ *such that*

25 $$ R_{2}R_{1}=\kappa(1-\theta)\operatorname{Id}+\kappa \theta N. $$

### Proof

Let $\theta_{i}=\frac{\beta_{i}}{{\alpha_{i}}+\beta_{i}}>0$, and observe that

26 $$ R_{i}=(\alpha_{i}+ \beta_{i}) \bigl((1-\theta_{i})\operatorname{Id}+ \theta_{i}N_{i} \bigr),\quad i\in\{1,2\}. $$

Next, let $\widetilde{N}_{2}=\frac{1}{\alpha_{1}+\beta_{1}}N_{2}\circ( \alpha_{1}+\beta_{1})\operatorname{Id}$, and note that $\widetilde{N}_{2}$ is nonexpansive. Now, set

27 $$ \widetilde{R}_{1}=(1-\theta_{1}) \operatorname{Id}+\theta_{1}N_{1},\qquad \widetilde{R}_{2}=(1- \theta_{2})\operatorname{Id}+\theta_{2} \widetilde{N}_{2}. $$

Then (26) and (27) yield

28a $$\begin{aligned} R_{2}R_{1}&= \bigl(({\alpha_{2}}+ \beta_{2}) \bigl((1-\theta_{2}){\mathrm{Id}}+ \theta_{2} N_{2}\bigr) \bigr) \bigl( ({ \alpha_{1}}+\beta_{1}) \bigl((1- \theta_{1}){ \mathrm{Id}}+\theta_{1} {N}_{1} \bigr) \bigr) \\ \end{aligned}$$

28b $$\begin{aligned} &=({\alpha_{1}}+\beta_{1}) ({\alpha_{2}}+ \beta_{2}) \biggl( \frac{1}{{\alpha_{1}}+\beta_{1}}\bigl((1-\theta_{2}){ \mathrm {Id}}+\theta_{2} {N}_{2}\bigr) \biggr) \bigl(({ \alpha_{1}}+\beta_{1}) \widetilde{R}_{1} \bigr) \\ \end{aligned}$$

28c $$\begin{aligned} &=({\alpha_{1}}+\beta_{1}) ({\alpha_{2}}+ \beta_{2})\widetilde{R}_{2} \widetilde{R}_{1}. \end{aligned}$$

We proceed by cases. Case I: $\alpha_{1}\alpha_{2}=0$. Observe that $0\in\{\alpha_{1},\alpha_{2}\}$ ⇔ $\max \{\frac{\beta_{1}}{{\alpha_{1}}+\beta_{1}} , \frac{\beta_{2}}{{\alpha_{2}}+\beta_{2}} \}=\max\{\theta_{1}, \theta_{2}\}=1$. The conclusion follows by observing that $\widetilde{R}_{i}$ is nonexpansive, $i\in\{1,2\}$.

Case II: $\alpha_{1}\alpha_{2}\neq0$. By assumption we must have $\frac{\beta_{1}}{{\alpha_{1}}+\beta_{1}} \frac{\beta_{2}}{{\alpha_{2}}+\beta_{2}}=\theta_{1}\theta_{2}<1$. We claim that $\widetilde{R}_{i}$, $i\in\{1,2\}$, satisfy the conditions of Theorem 3.3 with $(\alpha_{i},\beta _{i})$ replaced by $(1-\theta_{i},\theta_{i})$. Indeed, observe that $(1-\theta_{2})(1-\theta_{2}-1)\le\theta_{2}^{2} \Leftrightarrow \theta_{2}(\theta_{2}-1)\le\theta_{2}^{2} \Leftrightarrow\theta_{2}-1 \le\theta_{2}$, which is always true. Moreover, replacing $(\alpha_{i},\beta_{i})$ by $(1-\theta_{i},\theta_{i})$ yields $\delta_{1}=\frac{1-\theta_{1}}{\theta_{1}}$, $\delta_{2}=\frac{1-\theta_{2}}{\theta_{2}}$, $\delta_{3}=1$, and, consequently, $\delta_{4}= \frac{\theta_{2}(1-\theta_{1})+\theta_{1} (1-\theta_{2})}{(1-\theta_{1})(1-\theta_{2})}$. We claim that

29 $$ \theta_{1}+\theta_{2}-2 \theta_{1}\theta_{2}>0. $$

Indeed, recall that $\theta_{1}+\theta_{2}-2\theta_{1}\theta_{2} =\theta_{1}\theta_{2}( \frac{1}{\theta_{1}}+\frac{1}{\theta_{2}}-2) >\theta_{1}\theta_{2}( \frac{1}{\theta_{1}}+\theta_{1}-2) =\theta_{1}\theta_{2} ( \sqrt{\theta_{1}}-\frac{1}{\sqrt{\theta_{1}}} )^{2}>0$. This implies that $\delta_{1}+\delta_{2}= \frac{\theta_{1} +\theta_{2}-2\theta_{1}\theta_{2}}{\theta_{1}\theta_{2}}>0$. Moreover,

30 $$ \delta_{4}= \frac{(1-\theta_{1})(1-\theta_{2})}{\theta_{2}(1- \theta_{1})+\theta_{1}(1-\theta_{2})} = \frac{1-\theta_{1}-\theta_{2}+\theta_{1}\theta_{2}}{\theta_{1} +\theta_{2}-2\theta_{1}\theta_{2}} =-1+ \frac{1-\theta_{1}\theta_{2}}{\theta_{1}+\theta_{2}-2\theta _{1}\theta_{2}}>-1. $$

Therefore, by Theorem 3.3, we conclude that there exists a nonexpansive operator $N\colon X\to X$ such that $\widetilde{R}_{2}\widetilde{R}_{1}=\alpha\operatorname{Id}+\beta N$, $\alpha=\frac{\delta_{4}}{1+\delta_{4}} = \frac{1-\theta_{1}-\theta_{2}+\theta_{1}\theta_{2}}{1-\theta _{1}\theta_{2}} = \frac{{\alpha_{1}}{\alpha_{2}}}{ {\alpha_{1}}{\alpha_{2}}+{\alpha_{1}}\beta_{2}+{\alpha_{2}}\beta_{1}}$, and $\beta=\frac{1}{1+\delta_{4}} = \frac{\theta_{1}+\theta_{2}-2\theta_{1}\theta_{2}}{1-\theta _{1}\theta_{2}} = \frac{\beta_{1}{\alpha_{2}}+\beta_{2}{\alpha_{1}}}{ {\alpha_{1}}{\alpha_{2}}+{\alpha_{1}}\beta_{2}+{\alpha_{2}}\beta_{1}}$. Now combine with (28a)–(28c). □

## Applications to special cases

We start this section by recording the following simple lemma which can be easily verified, hence we omit the proof.

### Lemma 4.1

*Set* $(\widetilde{R}_{1},\widetilde{R}_{2})=(-R_{1},R_{2}\circ(- \operatorname{Id}))$. *Then the following hold*:

(i) $R_{2}R_{1}=\widetilde{R}_{2}\widetilde{R}_{1}$.
(ii) *Let* $\alpha_{i}>0$, *let* $\delta_{i}\in{\mathbb{R}}\smallsetminus\{0\}$, *and suppose that* $-\frac{1}{\delta_{i}}R_{i}$ *is* $\alpha_{i}$-*conically nonexpansive*. *Then* $\frac{1}{\delta_{i}}\widetilde{R}_{i}$ *is* $\alpha_{i}$-*conically nonexpansive*.

### Theorem 4.2

*Let* $i\in\{1,2\}$, *let* $\alpha_{i}>0$, *let* $\delta_{i}\in{\mathbb{R}}\smallsetminus\{0\}$, *let* $R_{i}\colon X\to X$ *be such that* $\frac{1}{\delta_{i}}R_{i}$ *is* $\alpha_{i}$-*conically nonexpansive*. *Suppose that either* $\alpha_{1}\alpha_{2}< 1$ *or* $\max\{\alpha_{1},\alpha_{2}\}=1$. *Set*

31 $$ \textstyle\begin{cases} \frac{\alpha_{1}+\alpha_{2}-2\alpha_{1}\alpha_{2}}{1-\alpha _{1}\alpha_{2}}, &\alpha_{1}\alpha_{2}< 1; \\ 1, &\max\{\alpha_{1},\alpha_{2}\}=1. \end{cases} $$

*Then there exists a nonexpansive operator* $N\colon X\to X$ *such that*

32 $$ R_{2}R_{1}=\delta_{1}\delta_{2} \bigl((1-\alpha)\operatorname{Id}+ \alpha N \bigr). $$

*Furthermore*, $\alpha<1 \Leftrightarrow[\alpha_{1}<1\textit{ and } \alpha_{2}<1]$.

### Proof

Set $(\widetilde{R}_{1},\widetilde{R}_{2})=(-R_{1},R_{2}\circ(- \operatorname{Id}))$ and set $R=R_{2}R_{1}$. The proof proceeds by cases.

Case I: $\delta_{i}>0$, $i\in\{1,2\}$. By assumption, there exist nonexpansive operators $N_{i}\colon X\to X$ such that $R_{i}=\delta_{i}(1-\alpha_{i})\operatorname{Id}+\delta_{i} \alpha_{i} N_{i}$. Moreover, one can easily check that $R_{i}$ satisfy the assumptions of Theorem 3.4 with $(\alpha_{i},\beta_{i})$ replaced by $(\delta_{i}(1-\alpha_{i}),\delta_{i}\alpha_{i})$. Applying Theorem 3.4, with $(\alpha_{i},\beta_{i})$ replaced by $(\delta_{i}(1-\alpha_{i}),\delta_{i}\alpha_{i})$, we learn that there exists a nonexpansive operator ${N}\colon X\to X$ such that $R=(\delta_{1}(1-\alpha_{1})+\delta_{1}\alpha_{1})(\delta_{2}(1- \alpha_{2})+\delta_{2}\alpha_{2}) ((1-\alpha)\operatorname{Id}+ \alpha{N}) =\delta_{1}\delta_{2}((1-\alpha)\operatorname{Id}+ \alpha{N})$, where

33 $$ \alpha= \frac{\delta_{1}(1-\alpha_{1})\delta_{2}\alpha_{2}+\delta _{2}(1-\alpha_{2})\delta_{1}\alpha_{1}}{ \delta_{1}(1-\alpha_{1})\delta_{2}\alpha_{2} +\delta_{2}(1-\alpha_{2})\delta_{1}\alpha_{1}+\delta_{1}(1-\alpha _{1})\delta_{2}(1-\alpha_{2})} = \frac{\alpha_{1}+\alpha_{2}-2\alpha_{1}\alpha_{2}}{1-\alpha _{1}\alpha_{2}}. $$

Finally, observe that $\alpha<1$ ⇔ [$\alpha_{1} \alpha_{2}<1$ and $\frac{\alpha_{1}+\alpha_{2}-2\alpha_{1}\alpha_{2}}{1-\alpha _{1}\alpha_{2}}<1$] ⇔ [$\alpha_{1}\alpha_{2}<1$ and $1-\alpha_{1}\alpha_{2}>\alpha_{1}+\alpha_{2}-2\alpha_{1}\alpha_{2}$] ⇔ [$\alpha_{1}\alpha_{2}<1$ and $(1-\alpha_{1})(1-\alpha_{2})>0$] ⇔ [$\alpha_{1}<1$ and $\alpha_{2}<1$].

Case II: $\delta_{i}<0$, $i\in\{1,2\}$. Observe that $\frac{1}{\delta_{i}}R_{i}=-\frac{1}{ \vert \delta_{i} \vert }R_{i}$ is $\alpha_{i}$-conically nonexpansive. Therefore, Lemma 4.1(ii), applied with $\delta _{i}$ replaced by $\vert \delta_{i} \vert $, implies that $\frac{1}{ \vert \delta_{i} \vert }\widetilde{R}_{i}$ are $\alpha_{i}$-conically nonexpansive. Now combine Lemma 4.1(i) and Case I applied with $(R_{i},\delta_{i})$ replaced by $(\widetilde{R}_{i}, \vert \delta_{i} \vert )$.

Case III: $\delta_{1}<0$ and $\delta_{2}>0$: Observe that $\frac{1}{\delta_{1}}R_{1}=-\frac{1}{ \vert \delta_{1} \vert }R_{1}$ is $\alpha_{1}$-conically nonexpansive. Now, using Lemma 4.1(i)&(ii), we have $-R=-R_{2}R_{1}=-\widetilde{R}_{2}\widetilde{R}_{1}$, and $-\frac{1}{\delta_{2}}\widetilde{R}_{2}$ is $\alpha_{2}$-conically nonexpansive. Now combine with Case II, applied with $(R_{1},R_{2},\delta_{1})$ replaced by $(\widetilde{R}_{1},-\widetilde{R}_{2}, \vert \delta_{1} \vert )$, to learn that there exists a nonexpansive mapping $N\colon X\to X$ such that $-R= \vert \delta_{1} \vert \delta_{2}((1-\alpha) \operatorname{Id}+\alpha N)$, and the conclusion follows.

Case IV: $\delta_{1}>0$ and $\delta_{2}<0$: Indeed, $-R=-R_{2}R_{1}$. Now combine with Case I applied with $R_{2}$ replaced by $-R_{2}$, in view of Lemma 4.1(ii). □

### Corollary 4.3

*Let* $\alpha\in\, ]0,1 [$, *let* $\beta>0$, *let* $\delta\in{\mathbb{R}}\smallsetminus\{0\}$, *let* $\{i,j\}=\{1,2\}$, *and suppose that* $\frac{1}{\delta}R_{i}$ *is* *α*-*averaged*, *and that* $R_{j}$ *is* $\frac{1}{\beta}$-*cocoercive*. *Set* $\overline{\alpha}=\frac{1}{2-\alpha}$. *Then* $\overline{\alpha}\in\, ]0,1 [$, *and there exists a nonexpansive operator* $N\colon X\to X$ *such that*

34 $$ R_{2}R_{1}=\beta\delta \bigl((1-\overline{\alpha}) \operatorname{Id}+ \overline{\alpha}N \bigr). $$

### Proof

Suppose first that $(i,j)=(1,2)$, and observe that there exists a nonexpansive operator *N̅* such that $R_{2}=\frac{\beta}{2}(\operatorname{Id}+\overline{N})$. Applying Theorem 4.7 with $m=2$, $(\alpha_{1},\alpha_{2},\delta_{1},\delta_{2})$ replaced by $(\alpha,1/2,\delta,\beta)$ yields that there exists a nonexpansive operator *N* such that $R_{2}R_{1}=\beta\delta ((1-\overline{\alpha})\operatorname{Id}+ \overline{\alpha} N )$, where

35 $$ \overline{\alpha} = \frac{\alpha+\frac{1}{2}-2\frac{\alpha}{2}}{1-\frac{\alpha}{2}} = \frac{1}{2-\alpha}\in\, ]0,1 [. $$

The case $(i,j)=(2,1)$ follows similarly. □

The assumption $\alpha_{1}\alpha_{2}<1$ is critical in the conclusion of Theorem 4.2 as we illustrate below.

### Example 4.4

($\alpha_{1}=\alpha_{2}>1$)

Let $\alpha>1$, and set $R_{1}=R_{2}=(1-\alpha){\mathrm{Id}}-\alpha{\mathrm{Id}}=(1-2\alpha ){\mathrm{Id}}$. Then

36 $$ R_{2}R_{1} = (1-2\alpha)^{2}{\mathrm{Id}}= \bigl(1-4\alpha+4\alpha^{2}\bigr) {\mathrm{Id}}. $$

Hence, $\operatorname{Id}-R_{2}R_{1}=4\alpha(1-\alpha)\operatorname{Id}$. That is, $\operatorname{Id}-R_{2}R_{1}$ is not monotone; hence, $R_{2}R_{1}$ is *not* conically nonexpansive by Lemma 2.9 applied with *T* replaced by $R_{2}R_{1}$.

The following proposition provides an abstract framework to construct a family of operators $R_{1}$ and $R_{2}$ such that $R_{1}$ is $\alpha_{1}$-conically nonexpansive, $R_{2}$ is $\alpha_{2}$-conically nonexpansive, $\alpha_{1}\alpha_{2}>1$, and the composition $R_{2}R_{1}$ fails to be conically nonexpansive.

### Proposition 4.5

*Let* $\theta\in{\mathbb{R}}$, *let* $\alpha_{1}>0$, *let* $\alpha_{2}>0$, *let*

37 $$ R_{\theta}= \begin{bmatrix} \cos\theta&-\sin\theta \\ \sin\theta&\cos\theta \end{bmatrix} , $$

*set*

38 $$ R_{1}=(1-\alpha_{1})\operatorname{Id}+ \alpha_{1} R_{\theta},\qquad R_{2}=(1- \alpha_{2})\operatorname{Id}-\alpha_{2} R_{\theta}, $$

*and set*

39 $$ \kappa=\alpha_{1}+\alpha_{2}-2 \alpha_{1}\alpha_{2}\sin^{2} \theta-( \alpha_{1}-\alpha_{2})\cos\theta. $$

*Then* $R_{1}$ *is* $\alpha_{1}$-*conically nonexpansive*, *and* $R_{2}$ *is* $\alpha_{2}$-*conically nonexpansive*. *Moreover*, *we have the implication* $\kappa<0$ ⇒ $R_{2}R_{1}$ *is* not *conically nonexpansive*.

### Proof

Set $S=R_{\pi/2}$, and observe that $S^{2}=-\operatorname{Id}$ and that $R_{\theta}=(\cos\theta) \operatorname{Id}+(\sin\theta) S$. Now,

40a $$\begin{aligned} R_{2}R_{1} &=\bigl((1-\alpha_{1}) \operatorname{Id}+\alpha_{1} R_{\theta}\bigr) \bigl((1- \alpha_{2})\operatorname{Id}-\alpha_{2} R_{\theta} \bigr) \end{aligned}$$

40b $$\begin{aligned} &=(1-\alpha_{1}-\alpha_{2}+\alpha_{1} \alpha_{2}) \operatorname{Id}+(\alpha_{1}- \alpha_{2})R_{\theta}-\alpha_{1} \alpha_{2} R_{2\theta} \end{aligned}$$

40c $$\begin{aligned} &=\bigl(1-\alpha_{1}-\alpha_{2}+\alpha_{1} \alpha_{2}+(\alpha_{1}- \alpha_{2})\cos \theta-\alpha_{1}\alpha_{2}\cos(2\theta) \bigr) \operatorname{Id} \\ &\quad {} +\bigl((\alpha_{1}-\alpha_{2})\sin\theta- \alpha_{1}\alpha_{2} \sin(2\theta)\bigr)S \end{aligned}$$

40d $$\begin{aligned} &=\bigl(1-\alpha_{1}-\alpha_{2}+\alpha_{1} \alpha_{2}+(\alpha_{1}- \alpha_{2})\cos \theta-\alpha_{1}\alpha_{2} \bigl(2\cos^{2} \theta-1\bigr) \bigr) \operatorname{Id} \\ &\quad {} +\bigl((\alpha_{1}-\alpha_{2})\sin\theta- \alpha_{1}\alpha_{2} \sin(2\theta)\bigr)S \end{aligned}$$

40e $$\begin{aligned} &=\bigl(1-\alpha_{1}-\alpha_{2}+2\alpha_{1} \alpha_{2}\sin^{2} \theta+( \alpha_{1}- \alpha_{2})\cos\theta\bigr)\operatorname{Id} \\ &\quad {}+\bigl(( \alpha_{1}- \alpha_{2})\sin\theta-\alpha_{1} \alpha_{2}\sin(2\theta)\bigr)S. \end{aligned}$$

Consequently,

41 $$\begin{aligned} \operatorname{Id}-R_{2}R_{1} ={}&\bigl( \alpha_{1}+\alpha_{2}-2\alpha_{1} \alpha_{2}\sin^{2} \theta-(\alpha_{1}- \alpha_{2})\cos\theta\bigr) \operatorname{Id} \\ &{}-\bigl(( \alpha_{1}-\alpha_{2})\sin\theta-\alpha_{1} \alpha_{2}\sin(2\theta)\bigr)S. \end{aligned}$$

Hence, $(\forall x\in{\mathbb{R}}^{2})$

42 $$ \bigl\langle (\operatorname {Id}-R_{2}R_{1})x \mid x \bigr\rangle =\bigl(\alpha_{1}+\alpha_{2}-2 \alpha_{1}\alpha_{2}\sin^{2} \theta-( \alpha_{1}-\alpha_{2}) \cos\theta\bigr) \Vert x \Vert ^{2}=\kappa \Vert x \Vert ^{2}. $$

Now, $R_{2}R_{1}$ is conically nonexpansive ⇒ $\operatorname{Id}-R_{2}R_{1}$ is monotone by Lemma 2.9, and the conclusion follows in view of (42). □

The following example provides two concrete instances where: (i) $\alpha_{1}>1$, $\alpha_{2}>1$, hence $\alpha_{1}\alpha_{2}>1$, (ii) $\alpha_{1}>1$, $\alpha_{2}<1$, $\alpha_{1}\alpha_{2}>1$. In both cases, $R_{2}R_{1} $ is *not* conically nonexpansive.

### Example 4.6

Suppose that one of the following holds:

(i) $\theta\in\, ]0,\pi/2 [$, $\epsilon\ge0$, $\delta\ge0$, $\alpha_{1}=\frac{1+\epsilon}{\sin^{2}\theta}$, and $\alpha_{2}=\frac{1+\delta}{\sin^{2}\theta}$.
(ii) $\theta\in\, ]\pi/4,\pi/2 [$, $\epsilon> \frac{\cos^{2}\theta(2-\cos^{2}\theta)}{(1-2\cos^{2}\theta )(1+\cos\theta)+\cos\theta}$, $\alpha_{1}=\frac{1+\epsilon}{\sin^{2}\theta}$, and $\alpha_{2}={\sin^{2}\theta}$.

Let $R_{\theta}$ be defined as in (37), let $R_{1}=(1-\alpha_{1})\operatorname{Id}+\alpha_{1} R_{\theta}$, and let $R_{2}=(1-\alpha_{2})\operatorname{Id}-\alpha_{2} R_{\theta}$. Then $\alpha_{1}\alpha_{2}>1$, and $R_{2}R_{1}$ is *not* conically nonexpansive.

### Proof

Let *κ* be defined as in (39). In view of Proposition 4.5, it is sufficient to show that $\kappa<0$. (i): Note that $\kappa<0$ ⇔ $\kappa\sin^{2} \theta<0$. Now,

43a $$\begin{aligned} \kappa\sin^{2} \theta&=2+\epsilon+\delta-(\epsilon-\delta) \cos \theta-2-2 \epsilon-2\delta-2 \epsilon\delta \end{aligned}$$

43b $$\begin{aligned} &=-\bigl(\epsilon(1+\cos\theta)+\delta(1-\cos\theta)+2 \epsilon \delta\bigr)< 0. \end{aligned}$$

(ii): We have

44a $$\begin{aligned} \kappa&=\frac{1+\epsilon+\sin^{4}\theta}{\sin^{2}\theta}-2(1+ \epsilon)\sin^{2}\theta- \frac{1+\epsilon-\sin^{4}\theta}{\sin^{2}\theta}\cos\theta \end{aligned}$$

44b $$\begin{aligned} &=-\frac{1}{\sin^{2}\theta} \bigl(2(1+\epsilon)\sin^{4}\theta-\bigl(1+ \epsilon+\sin^{4}\theta\bigr)+\bigl(1+\epsilon-\sin^{4} \theta\bigr)\cos \theta \bigr) \end{aligned}$$

44c $$\begin{aligned} &=-\frac{1}{1-\cos^{2}\theta} \bigl(\bigl(2\sin^{4}\theta+\cos\theta-1 \bigr) \epsilon+\sin^{4}\theta(1-\cos\theta) -(1-\cos\theta) \bigr) \end{aligned}$$

44d $$\begin{aligned} &=-\frac{1-\cos\theta}{1-\cos^{2}\theta} \bigl(\bigl(2(1+\cos\theta) \bigl(1- \cos^{2}\theta\bigr)-1\bigr)\epsilon+1-2\cos^{2}\theta+ \cos^{4}\theta-1 \bigr) \end{aligned}$$

44e $$\begin{aligned} &=-\frac{1}{1+\cos\theta} \bigl(\bigl(1+2\cos\theta-2\cos^{2}\theta-2 \cos^{3} \theta\bigr)\epsilon-\cos^{2}\theta\bigl(2- \cos^{2}\theta\bigr) \bigr) \end{aligned}$$

44f $$\begin{aligned} &=-\frac{1}{1+\cos\theta} \bigl(\bigl(1-2\cos^{2}\theta\bigr) (1+\cos \theta)+ \cos\theta\bigr)\epsilon-\cos^{2}\theta\bigl(2- \cos^{2}\theta\bigr) ). \end{aligned}$$

Now, observe that $(\forall\theta\in\, ]\frac{\pi}{4},\frac{\pi}{2} [ )$ $1-2\cos^{2}\theta=-\cos(2\theta) >0$. Consequently, $(1-2\cos^{2}\theta)(1+\cos\theta)+\cos\theta>\cos\theta>0$. Now use the assumption $\epsilon> \frac{\cos^{2}\theta(2-\cos^{2}\theta)}{(1-2\cos^{2}\theta )(1+\cos\theta)+\cos\theta}$ to learn that $(1-2\cos^{2}\theta)(1+\cos\theta)+\cos\theta)\epsilon-\cos^{2} \theta(2-\cos^{2}\theta)>0$, hence $\kappa<0$, and the conclusion follows. □

### Theorem 4.7

(composition of *m* scaled conically nonexpansive operators)

*Let* $m\ge2$ *be an integer*, *set* $I=\{1,\ldots,m\}$, *let* $(R_{i})_{i\in I}$ *be a family of operators from* *X* *to* *X*, *let* $r\in I$, *let* $\alpha_{i}$ *be real numbers such that* $\{ {\alpha_{i}} \mid{i\in I\smallsetminus\{r\} } \}\subseteq ]0,1 [$ *and* $\alpha_{r}>0$, *let* $\delta_{i}$ *be real numbers in* ${\mathbb{R}}\smallsetminus\{0\}$, *and suppose that*, *for every* $i\in I$, $\frac{1}{\delta_{i}}R_{i}$ *is* $\alpha_{i}$-*conically nonexpansive*. *Set*

45 $$ \overline{\alpha}= \frac{\sum_{\substack{i=1 \\ i\neq {r}}}^{m} \frac{\alpha_{i}}{1-\alpha_{i}} }{1+\sum_{\substack{i=1 \\ i\neq {r}}}^{m}\frac{\alpha _{i}}{1-\alpha_{i}}}. $$

*Suppose that* $\alpha_{r}\overline{\alpha}<1$, *and set*

46 $$ \alpha= \textstyle\begin{cases} \frac{\sum_{i=1 }^{m} \frac{\alpha_{i}}{1-\alpha_{i}} }{1+\sum_{i=1 }^{m}\frac{\alpha_{i}}{1-\alpha_{i}}}, & \alpha_{r} \neq1; \\ 1, & \alpha_{r}=1. \end{cases} $$

*Then there exists a nonexpansive operator* $N\colon X\to X$ *such that*

47 $$ R_{m}\cdots R_{1}=\delta_{m}\cdots \delta_{1}\bigl((1-\alpha) \operatorname{Id}+\alpha N\bigr). $$

### Proof

First, observe that $(\forall i\in I\smallsetminus\{r\})$, $\frac{1}{\delta_{i}}R_{i}$ is nonexpansive. If $\alpha_{r}=1$, then $(\forall i\in\{1,\ldots,m\})$ $R_{i}$ is ${ \vert \delta_{i} \vert }$-Lipschitz continuous and the conclusion readily follows. Now, suppose that $\alpha_{r}\neq1$. We proceed by induction on $k\in\{2,\ldots,m\}$. At $k=2$, the claim holds by Theorem 4.2. Now, suppose that the claim holds for some $k\in\{2,\ldots,m-1\}$. Let $(R_{i})_{1\le i\le k+1}$ be a family of operators from *X* to *X*, let $\overline{r}\in\{1,\ldots,k,k+1\}$, let $\alpha_{i}$ be real numbers such that $\{ {\alpha_{i}} \mid{i\in\{1,\ldots,k,k+1\}\smallsetminus\{ \overline{r}\} } \}\subseteq ]0,1 [$ and $\alpha_{\overline{r}} \in\, ]0,+\infty [\smallsetminus \{1\}$, let $\delta_{i}$ be real numbers in ${\mathbb{R}}\smallsetminus\{0\}$, and suppose that, for every $i\in\{1,\ldots,k+1\}$, $\frac{1}{\delta_{i}}R_{i}$ is $\alpha_{i}$-conically nonexpansive. Set $\overline{\beta}= \frac{\sum_{\substack{i=1 \\ i\neq\overline{r}}}^{k+1} \frac{\alpha_{i}}{1-\alpha_{i}} }{1+\sum_{\substack{i=1 \\ i\neq\overline{r}}}^{k+1}\frac{\alpha _{i}}{1-\alpha_{i}}}$, and suppose that $\alpha_{\overline{r}}\overline{\beta}<1$. We examine two cases.

Case I: $\alpha_{k+1}=\alpha_{\overline{r}}$. In this case the conclusion follows by applying Theorem 4.2 in view of the inductive hypothesis with $(R_{1},R_{2})$ replaced by $(R_{k}\ldots R_{1},R_{k+1})$ and $(\delta_{1},\delta_{2},\alpha_{1},\alpha_{2})$ replaced by $(\delta_{1}\ldots\delta_{k},\delta_{k+1}, ({\sum_{i=1}^{k} \frac{\alpha_{i}}{1-\alpha_{i}}} )/ ({1+\sum_{i=1}^{k} \frac{\alpha_{i}}{1-\alpha_{i}}} ), \alpha_{k+1})$.

Case II: $\alpha_{k+1}\neq\alpha_{\overline{r}}$. We claim that

48 $$ \alpha_{k+1} \frac{\sum_{i=1}^{k}\frac{\alpha_{i}}{1-\alpha_{i}}}{ 1+\sum_{i=1}^{k}\frac{\alpha_{i}}{1-\alpha_{i}}}< 1. $$

To this end, set $\hat{\alpha} = \frac{\sum_{\substack{i=1 \\ i\neq\overline{r}}}^{k}\frac{\alpha _{i}}{1-\alpha_{i}}}{ 1+\sum_{\substack{i=1 \\ i\neq\overline{r}}}^{k}\frac{\alpha _{i}}{1-\alpha_{i}}}$, and observe that $\hat{\alpha}<\overline{\beta}$. By assumption we have $\alpha_{\overline{r}}\overline{\beta}<1$. Altogether, we conclude that $\alpha_{\overline{r}}\hat{\alpha}<1$. It follows from the inductive hypothesis that

49 $$ \text{$\frac{1}{\delta_{1}\ldots\delta_{k}}(R_{k}\ldots R_{1})$ is $ \frac{\sum_{i=1}^{k}\frac{\alpha_{i}}{1-\alpha_{i}}}{ 1+\sum_{i=1}^{k}\frac{\alpha_{i}}{1-\alpha_{i}}}$-conically nonexpansive}. $$

Next note that

50a $$\begin{aligned} \frac{\sum_{i=1}^{k}\frac{\alpha_{i}}{1-\alpha_{i}}}{ 1+\sum_{i=1}^{k}\frac{\alpha_{i}}{1-\alpha_{i}}} & = \frac{\frac{\sum_{\substack{i=1 \\ i\neq\overline{r}}}^{k}\frac {\alpha_{i}}{1-\alpha_{i}}+\frac{\alpha_{\overline{r}}}{1-\alpha _{\overline{r}}}}{1+\sum_{\substack{i=1 \\ i\neq\overline {r}}}^{k}\frac{\alpha_{i}}{1-\alpha_{i}}}}{\frac{1+\sum_{\substack {i=1 \\ i\neq\overline{r}}}^{k}\frac{\alpha_{i}}{1-\alpha _{i}}+\frac{\alpha_{\overline{r}}}{1-\alpha_{\overline {r}}}}{1+\sum_{\substack{i=1 \\ i\neq\overline{r}}}^{k}\frac {\alpha_{i}}{1-\alpha_{i}}}} = \frac{\hat{\alpha}+\frac{\alpha_{\overline{r}}}{ (1-\alpha_{\overline{r}}) (1+\sum_{\substack{i=1 \\ i\neq \overline{r}}}^{k}\frac{\alpha_{i}}{1-\alpha_{i}} )}}{1+\frac {\alpha_{\overline{r}}}{(1-\alpha_{\overline{r}}) (1+\sum_{\substack{i=1 \\ i\neq\overline{r}}}^{k}\frac{\alpha _{i}}{1-\alpha_{i}} )}} \end{aligned}$$

50b $$\begin{aligned} &= \frac{\hat{\alpha}(1-\alpha_{\overline{r}}) (1+\sum_{\substack{i=1 \\ i\neq\overline{r}}}^{k}\frac{\alpha _{i}}{1-\alpha_{i}} )+\alpha_{\overline{r}}}{(1-\alpha _{\overline{r}}) (1+\sum_{\substack{i=1 \\ i\neq\overline{r}}}^{k}\frac {\alpha_{i}}{1-\alpha_{i}} )+\alpha_{\overline{r}}} \end{aligned}$$

50c $$\begin{aligned} &= \frac{\alpha_{\overline{r}} (1-\hat{\alpha} (1+\sum_{\substack{i=1 \\ i\neq\overline{r}}}^{k}\frac {\alpha_{i}}{1-\alpha_{i}} ) )+\hat{\alpha} (1+\sum_{\substack{i=1 \\ i\neq\overline{r}}}^{k}\frac {\alpha_{i}}{1-\alpha_{i}} )}{1+(1-\alpha_{\overline{r}})\sum_{\substack{i=1 \\ i\neq\overline{r}}}^{k}\frac{\alpha _{i}}{1-\alpha_{i}}}. \end{aligned}$$

Because $\alpha_{\overline{r}}\overline{\beta}<1$, we learn that $1+(1-\alpha_{\overline{r}})\sum_{ \substack{i=1 \\ i\neq\overline{r}}}^{k} \frac{\alpha_{i}}{1-\alpha_{i}}>0$. Moreover, because $\hat{\alpha}<1$, we have $\alpha_{k+1}\hat{\alpha}<1$. Therefore, (50a)–(50c) implies

51a $$\begin{aligned} & \alpha_{k+1} \frac{\sum_{i=1}^{k}\frac{\alpha_{i}}{1-\alpha_{i}}}{1 +\sum_{i=1}^{k}\frac{\alpha_{i}}{1-\alpha_{i}}}< 1 \end{aligned}$$

51b $$\begin{aligned} &\quad \Leftrightarrow\quad \alpha_{k+1} \Biggl(\alpha_{\overline{r}} \Biggl(1- \hat{\alpha} \Biggl(1 +\sum_{\substack{i=1 \\ i\neq\overline{r}}}^{k} \frac{\alpha_{i}}{1-\alpha_{i}} \Biggr) \Biggr) +\hat{\alpha} \Biggl(1 + \sum _{\substack{i=1 \\ i\neq\overline{r}}}^{k} \frac{\alpha_{i}}{1-\alpha_{i}} \Biggr) \Biggr) \\ &\hphantom{\quad \Leftrightarrow\quad}\quad < 1+(1-\alpha_{ \overline{r}}) \sum_{\substack{i=1 \\ i\neq\overline{r}}}^{k} \frac{\alpha_{i}}{1-\alpha_{i}} \end{aligned}$$

51c $$\begin{aligned} &\quad \Leftrightarrow\quad \alpha_{\overline{r}} \Biggl(\alpha_{k+1} \Biggl(1- \hat{\alpha} \Biggl(1+\sum_{\substack{i=1 \\ i\neq\overline{r}}}^{k} \frac{\alpha_{i}}{1-\alpha_{i}} \Biggr) \Biggr) +\sum_{ \substack{i=1 \\ i\neq\overline{r}}}^{k} \frac{\alpha_{i}}{1-\alpha_{i}} \Biggr) \\ &\hphantom{\quad \Leftrightarrow\quad}\quad < \Biggl(1+\sum_{ \substack{i=1 \\ i\neq\overline{r}}}^{k} \frac{\alpha_{i}}{1-\alpha_{i}} \Biggr) (1-\alpha_{k+1}\hat{\alpha}) \end{aligned}$$

51d $$\begin{aligned} &\quad \Leftrightarrow\quad \alpha_{\overline{r}} \Biggl(\alpha_{k+1} \Biggl(1- \sum_{\substack{i=1 \\ i\neq\overline{r}}}^{k} \frac{\alpha_{i}}{1-\alpha_{i}} \Biggr) +\sum_{ \substack{i=1 \\ i\neq\overline{r}}}^{k} \frac{\alpha_{i}}{1-\alpha_{i}} \Biggr) \\ &\hphantom{\quad \Leftrightarrow\quad}\quad < \Biggl(1+\sum_{ \substack{i=1 \\ i\neq\overline{r}}}^{k} \frac{\alpha_{i}}{1-\alpha_{i}} \Biggr) (1-\alpha_{k+1}\hat{\alpha}) \end{aligned}$$

51e $$\begin{aligned} &\quad \Leftrightarrow\quad \alpha_{\overline{r}} \frac{\alpha_{k+1} (1-\sum_{\substack{i=1 \\ i\neq\overline{r}}}^{k} \frac{\alpha_{i}}{1-\alpha_{i}} )+\sum_{\substack{i=1 \\ i\neq\overline{r}}}^{k}\frac{\alpha_{i}}{1-\alpha_{i}}}{ (1+\sum_{\substack{i=1 \\ i\neq\overline{r}}}^{k}\frac {\alpha_{i}}{1-\alpha_{i}} )(1-\alpha_{k+1}\hat{\alpha})}< 1. \end{aligned}$$

Now, observe that

52 $$\begin{aligned} \alpha_{k+1} \Biggl(1-\sum _{\substack{i=1 \\ i\neq\overline{r}}}^{k} \frac{\alpha_{i}}{1-\alpha_{i}} \Biggr) +\sum _{ \substack{i=1 \\ i\neq\overline{r}}}^{k} \frac{\alpha_{i}}{1-\alpha_{i}} &= \Biggl(\sum_{ \substack{i=1 \\ i\neq\overline{r}}}^{k} \frac{\alpha_{i}}{1-\alpha_{i}}+ \frac{\alpha_{k+1}}{1-\alpha_{k+1}} \Biggr) (1-\alpha_{k+1}) \\ & = \sum_{ \substack{i=1 \\ i\neq\overline{r}}}^{k+1} \frac{\alpha_{i}}{1-\alpha_{i}}(1- \alpha_{k+1}) \end{aligned}$$

and

53a $$\begin{aligned} \Biggl(1+\sum_{\substack{i=1 \\ i\neq\overline{r}}}^{k} \frac{\alpha_{i}}{1-\alpha_{i}} \Biggr) (1-\alpha_{k+1} \hat{\alpha} ) &=1+\sum _{\substack{i=1 \\ i\neq\overline{r}}}^{k} \frac{\alpha_{i}}{1-\alpha_{i}} - \alpha_{k+1}\sum_{ \substack{i=1 \\ i\neq\overline{r}}}^{k} \frac{\alpha_{i}}{1-\alpha_{i}} \\ \end{aligned}$$

53b $$\begin{aligned} &= \Biggl(1+\sum_{\substack{i=1 \\ i\neq\overline{r}}}^{k} \frac{\alpha_{i}}{1-\alpha_{i}}+ \frac{\alpha_{k+1}}{1-\alpha_{k+1}} \Biggr) (1-\alpha_{k+1}) \end{aligned}$$

53c $$\begin{aligned} &= \Biggl(1+\sum_{\substack{i=1 \\ i\neq\overline{r} }}^{k+1} \frac{\alpha_{i}}{1-\alpha_{i}} \Biggr) (1-\alpha_{k+1}). \end{aligned}$$

In view of (52) and (53a)–(53c), (51a)–(51e) becomes

54 $$ \alpha_{k+1} \frac{\sum_{i=1}^{k}\frac{\alpha_{i}}{1-\alpha_{i}}}{1+\sum_{i=1}^{k}\frac{\alpha_{i}}{1-\alpha_{i}}}< 1\quad \Leftrightarrow\quad \alpha_{\overline{r}} \frac{\sum_{i=1}^{k+1}\frac{\alpha_{1}}{1-\alpha_{i}}}{1+\sum_{i=1}^{k+1}\frac{\alpha_{1}}{1-\alpha_{i}}}= \alpha_{\overline{r}}\overline{ \beta}< 1. $$

This proves (48). Now proceed similar to Case I in view of (48) and (49). □

The assumption $\alpha_{r}\overline{\alpha}<1$ is critical in the conclusion of the above theorem as we illustrate in the following example.

### Example 4.8

Let $\epsilon>0$, let $\delta>1$, let $\alpha_{1}\in\, ]0,\frac{1}{2}(\sqrt{(\epsilon+\delta)^{2}+4}-( \epsilon+\delta))[$, let $\alpha_{2}=\alpha_{1}+\delta+\epsilon$, and let

55 $$ S= \begin{bmatrix} 0&-1 \\ 1&0 \end{bmatrix} . $$

Set $R_{1}=(1-\alpha_{1})\operatorname{Id}-\alpha_{1} S$, $R_{2}=(1-\alpha_{2})\operatorname{Id}+\alpha_{2} S$, $R_{3}=-\frac{1}{\delta}S$, and

56 $$ R=R_{3}R_{2}R_{1}. $$

Then $R=R_{3}R_{1}R_{2}=R_{1}R_{2}R_{3}=R_{1}R_{3}R_{2}=R_{2}R_{3}R_{1}=R_{2}R_{1}R_{3}$. Moreover, the following hold:

(i) $\alpha_{1}\in\, ]0,1[$, $\alpha_{2}>1$, and $\alpha_{1}\alpha_{2}<1$.
(ii) $R_{3}$ is $\alpha_{3}$-conically nonexpansive where $\alpha_{3}=\frac{1+\delta}{2\delta}\in\, ]1/2,1]$.
(iii) $\frac{\alpha_{1}+\alpha_{2}-2\alpha_{1}\alpha_{2}}{1-\alpha _{1}\alpha_{2}} \alpha_{3}>1$.
(iv) $R= (\frac{\epsilon+\delta}{\delta} )\operatorname {Id}+ ( \frac{\alpha_{1}+\alpha_{2}-2\alpha_{1}\alpha_{2}-1}{\delta} )S$.
(v) $\operatorname{Id}-R=-\frac{\epsilon}{\delta}\operatorname{Id}- (\frac{\alpha_{1}+\alpha_{2}-2\alpha\alpha_{2}-1}{\delta} )S$. Hence, $\operatorname{Id}-R$ is not monotone.
(vi) *R* is *not* conically nonexpansive.

### Proof

It is straightforward to verify that $R=R_{3}R_{1}R_{2}=R_{1}R_{2}R_{3}=R_{1}R_{3}R_{2}=R_{2}R_{3}R_{1}=R_{2}R_{1}R_{3}$. (i): It is clear that $\alpha_{1}\in\, ]0,1[$ and that $\alpha_{2}>1$. Note that $\alpha_{1}\alpha_{2}<1 \Leftrightarrow\alpha_{1}^{2}+(\epsilon+ \delta)\alpha_{1}-1<0$ ⇔ $\alpha_{1}$ lies between the roots of the quadratic $x^{2}+(\epsilon+\delta)x-1$, and the conclusion follows from the quadratic formula. (ii): This follows from [2, Proposition 4.38]. (iii): Indeed, in view of (i) we have

57a $$\begin{aligned} & \frac{\alpha_{1}+\alpha_{2} -2\alpha_{1}\alpha_{2}}{1-\alpha_{1}\alpha_{2}}\alpha_{3}>1 \\ &\quad \Leftrightarrow\quad (\alpha_{1}+\alpha_{2} -2 \alpha_{1}\alpha_{2}) \alpha_{3}>1- \alpha_{1}\alpha_{2} \end{aligned}$$

57b $$\begin{aligned} &\quad \Leftrightarrow\quad (\alpha_{1}+\alpha_{2}-2 \alpha_{1}\alpha_{2}) (1+ \delta) >2(1- \alpha_{1}\alpha_{2})\delta \end{aligned}$$

57c $$\begin{aligned} &\quad \Leftrightarrow\quad (\alpha_{1}+\alpha_{2}) (1+\delta) -2 \alpha_{1} \alpha_{2}-2\alpha_{1} \alpha_{2}\delta>2\delta-2\alpha_{1} \alpha_{2}\delta \end{aligned}$$

57d $$\begin{aligned} &\quad \Leftrightarrow\quad (\alpha_{1}+\alpha_{2}) (1+\delta) -2 \alpha_{1} \alpha_{2} >2\delta \end{aligned}$$

57e $$\begin{aligned} &\quad \Leftrightarrow\quad (2\alpha_{1}+\epsilon+\delta) (1+\delta) -2 \alpha_{1}(\alpha_{1}+\epsilon+\delta) >2\delta \end{aligned}$$

57f $$\begin{aligned} &\quad \Leftrightarrow\quad 2\alpha_{1}(1+\delta-\alpha_{1}- \epsilon- \delta) +\delta^{2}+\delta(1+\epsilon) +\epsilon>2\delta \end{aligned}$$

57g $$\begin{aligned} &\quad \Leftrightarrow\quad 2\alpha_{1}(\alpha_{1}-1+\epsilon)< \delta^{2}- \delta+\epsilon\delta+\epsilon=\delta^{2}- \delta+(1+\delta) \epsilon. \end{aligned}$$

Now, because $\alpha_{1}<1$, $\delta\ge1$, we learn that $2\alpha_{1}(\alpha_{1}-1+\epsilon)<2\alpha_{1}\epsilon<(1+ \delta)\epsilon<(1+\delta)\epsilon+\delta^{2}-\delta$, and the conclusion follows. (iv): It is straightforward, by noting that $S^{2}=-\operatorname{Id}$, to verify that $R_{2}R_{1}=R_{1}R_{2}=(1-\alpha_{1}-\alpha_{2}+\alpha_{1}\alpha_{2}) \operatorname{Id}+ (\alpha_{2}(1-\alpha_{1})-\alpha_{1}(1-\alpha_{2}))S- \alpha_{1}\alpha_{2}S^{2} =(1-\alpha_{1}-\alpha_{2}+2\alpha_{1} \alpha_{2})\operatorname{Id}+(\alpha_{2}-\alpha_{1})S $. Consequently, $R_{3}R_{2}R_{1}=\frac{1}{\delta}(-(1-\alpha_{1}-\alpha_{2}+2 \alpha_{1}\alpha_{2})S -(\alpha_{2}-\alpha_{1})S^{2}) = \frac{1}{\delta}((\alpha_{2}-\alpha_{1})\operatorname{Id}-(1- \alpha_{1}-\alpha_{2}+2\alpha_{1}\alpha_{2})S) = \frac{\epsilon+\delta}{\delta}\operatorname{Id}+ \frac{\alpha_{1}+\alpha_{2}-2\alpha_{1}\alpha_{2}-1}{\delta}S$. (v): This is a direct consequence of (iv). (vi): Combine (v) and Lemma 2.9. □

### Theorem 4.9

(Composition of cocoercive operators)

*Let* $m\ge1$ *be an integer*, *set* $I=\{1,\ldots,m\}$, *let* $(R_{i})_{i\in I}$ *be a family of operators from* *X* *to* *X*, *let* $\beta_{i}$ *be real numbers in* $]0,+\infty [$, *and suppose that*, *for every* $i\in I$, $R_{i}$ *is* $\frac{1}{\beta_{i}}$-*cocoercive*. *Then there exists a nonexpansive operator* $N\colon X\to X$ *such that*

58 $$ R_{m}\cdots R_{1}=\beta_{m}\cdots \beta_{1} \biggl(\frac{1}{1+m} \operatorname{Id}+ \frac{m}{1+m} N \biggr). $$

### Proof

Apply Theorem 4.7 with $(\alpha_{i},\delta_{i})$ replaced by $(1/2,\beta_{i})$, $i\in\{1,\ldots,m\}$. □

## Application to the Douglas–Rachford algorithm

### Theorem 5.1

(Averagedness of the Douglas–Rachford operator)

*Let* $\mu>\omega\ge0$, *and let* $\gamma\in\, ]0, {(\mu-\omega)}/{(2\mu\omega}) [$. *Suppose that one of the following holds*:

(i) *A* *is maximally* $(-\omega) $-*monotone and* *B* *is maximally* *μ*-*monotone*.
(ii) *A* *is maximally* *μ*-*monotone and* *B* *is maximally* $(-\omega) $-*monotone*.

*Set*

59 $$ T=\frac{1}{2}(\operatorname{Id}+ R_{\gamma B}R_{\gamma A}),\quad \textit{and}\quad \alpha= \frac{\mu-\omega}{2(\mu-\omega-\gamma\mu\omega)}. $$

*Then* $\alpha\in\, ]0,1 [$ *and* *T* *is* *α*-*averaged*.

### Proof

Suppose that (i) holds. Note that *γA* is $-\gamma\omega$-monotone, and

60 $$ -\gamma\omega>-\frac{\mu-\omega}{2\mu} \ge- \frac{\mu}{2\mu }>- 1. $$

Using (13) and Fact 2.1 we learn that $J_{\gamma A}$ and, in turn, *T* are single-valued and $\operatorname{dom}J_{\gamma A}=\operatorname{dom}T=X$. It follows from [3, Proposition 4.3 and Table 1] that $-R_{\gamma A}$ is $\frac{1}{1+\gamma\mu}$-conically nonexpansive and $-R_{\gamma B}$ is $\frac{1}{1-\gamma\omega}$-conically nonexpansive. It follows from Theorem 4.2, applied with $(\alpha_{1},\beta_{1},\delta_{1},\alpha_{2},\beta_{2},\delta_{2})$ replaced by $(1-\frac{1}{1+\gamma\mu},\frac{1}{1+\gamma\mu},-1, 1- \frac{1}{1-\gamma\omega},\frac{1}{1-\gamma\omega},-1)$, that $R_{\gamma B} R_{\gamma A}$ is $\frac{\mu-\omega}{\mu-\omega-\gamma\mu\omega}$-conically nonexpansive. Therefore, there exists a nonexpansive mapping $N\colon X\to X$ such that

61 $$ R_{\gamma B}R_{\gamma A}=(1-\delta)\operatorname{Id}+\delta N,\quad \delta=\frac{\mu-\omega}{ \mu-\omega-\gamma\mu\omega}. $$

The conclusion now follows by applying Proposition 2.8 with $(\beta,N) $ replaced by $(\frac{\alpha}{\delta}, R_{\gamma B}R_{\gamma A})$. Finally, notice that $\gamma<\frac{\mu-\omega}{2\mu\omega}$, which implies that $0<\mu-\omega< 2(\mu-\omega-\gamma\mu\omega)$. Therefore,

62 $$ \alpha =\frac{\mu-\omega}{2(\mu-\omega-\gamma\mu\omega) } \in\, ]0,1 [. $$

The proof of (ii) follows similarly. □

### Corollary 5.2

([9, Theorem 4.5(ii)])

*Let* $\mu>\omega\ge0$, *and let* $\gamma\in\, ]0, {(\mu-\omega)}/{(2\mu\omega)} [$. *Suppose that one of the following holds*:

(i) *A* *is maximally* $(-\omega) $-*monotone and* *B* *is maximally* *μ*-*monotone*.
(ii) *A* *is maximally* *μ*-*monotone and* *B* *is maximally* $(-\omega) $-*monotone*.

*Set* $T=\frac{1}{2}(\operatorname{Id}+ R_{\gamma B}R_{\gamma A}) $ *and let* $x_{0}\in X$. *Then* $(\exists \overline{x}\in\operatorname{Fix}T=\operatorname{Fix}R_{ \gamma B}R_{\gamma A})$ *such that* $T^{n} x_{0}\,\rightharpoonup \,\overline{x}$.

### Proof

Combine Theorem 5.1 and [2, Theorem 5.15]. □

### Remark 5.3

In view of (13), one might think that the scaling factor *γ* is required *only* to guarantee the single-valuedness and the full domain of *T*. However, it is actually critical to guarantee convergence as well, as we illustrate in Example 5.4.

### Example 5.4

Let $\mu>\omega\ge0$, let *U* be a closed linear subspace of *X*, suppose that^9^

63 $$ A=N_{U}+\mu\operatorname{Id},\qquad B=-\omega\operatorname{Id}. $$

Then *A* is *μ*-monotone, *B* is −*ω*-monotone, and $(\forall\gamma\in[{1}/{(2\omega)},{1}/{\omega}[)$ $J_{\gamma B}$ is single-valued. Furthermore, we have

64 $$ T=\frac{1}{2} (\operatorname{Id}+R_{\gamma B}R_{\gamma A}) = \frac{1+\gamma\omega}{(1-\gamma\omega)(1+\gamma\mu)}P_{U} - \frac{\gamma\omega}{1-\gamma\omega} \operatorname{Id}, $$

and $(\forall x_{0}\in U^{\perp})$ $(T^{n} x_{0})_{n\in{\mathbb {N}}}$ does not converge.

### Proof

Indeed, one can verify that

65 $$ J_{\gamma A}=\frac{1}{1-\gamma\omega}\operatorname{Id},\qquad J_{ \gamma B}= \frac{1}{1+\gamma\mu}P_{U}. $$

Consequently,

66 $$ R_{\gamma A}=\frac{1+\gamma\omega}{1-\gamma\omega} \operatorname{Id},\qquad R_{\gamma B}= \frac{2}{1+\gamma\mu}P_{U}- \operatorname{Id}, $$

and (64) follows. Therefore,

67 $$ T_{ |U^{\perp}}= -\frac{\gamma\omega}{1-\gamma\omega} \operatorname{Id}\quad \text{and}\quad {-} \frac{\gamma\omega}{1-\gamma\omega}\in\, ]{-}\infty,-1 ]. $$

Hence, $(\forall x_{0}\in U^{\perp})$ $(T^{n} x_{0})_{n\in{\mathbb{N}}}$ does not converge. □

Before we proceed to the convergence analysis, we recall that if *T* is averaged and $\operatorname{Fix}T\neq\varnothing$ then $(\forall x\in X)$ we have (see, e.g., [22, Theorem 3.7])

68 $$ T^{n} x-T^{n+1}x\to0. $$

We conclude this section by proving the strong convergence of the shadow sequence of the Douglas–Rachford algorithm.

### Theorem 5.5

(Convergence analysis of the Douglas–Rachford algorithm)

*Let* $\mu>\omega\ge0$, *and let* $\gamma\in\, ]0, {(\mu-\omega)}/{(2\mu\omega)} [$. *Suppose that one of the following holds*:

(i) *A* *is maximally* *μ*-*monotone and* *B* *is maximally* $(-\omega) $-*monotone*.
(ii) *A* *is maximally* $(-\omega)$-*monotone and* *B* *is maximally* *μ*-*monotone*.

*Set*

69 $$ T=\frac{1}{2}(\operatorname{Id}+ R_{\gamma B}R_{\gamma A}), $$

*and let* $x_{0}\in X$. *Then* $\operatorname{zer}(A+B)\neq\varnothing$. *Moreover*, *there exist* $\overline{x}\in\operatorname{Fix}T =\operatorname{Fix}R_{\gamma B}R_{ \gamma A}$, $\operatorname{zer}(A+B)=\{J_{\gamma A}\overline{x}\} =\{J_{\gamma B} R_{\gamma A} \overline{x}\}$, $T^{n}x_{0}\,\rightharpoonup \,\overline{x}$, $J_{\gamma A}T^{n}x_{0}\to J_{\gamma A}\overline{x}$, *and* $J_{\gamma B} R_{\gamma A}T^{n}x_{0}\to J_{\gamma B} R_{\gamma A} \overline{x}$.

### Proof

Suppose that (i) holds. Since $A+B$ is $(\mu-\omega)$-monotone and $\mu-\omega>0$, we conclude from [2, Proposition 23.35] that $\operatorname{zer}(A+B)$ is a singleton. Combining with Fact 2.1 with $(A,B)$ replaced by $(\gamma A,\gamma B)$ yields $\operatorname{zer}(A+B)=\operatorname{zer}(\gamma A+\gamma B) =\{J_{ \gamma A}\overline{x}\} =\{J_{\gamma B} R_{\gamma A} \overline{x}\}$. The claim that $T^{n}x_{0}\,\rightharpoonup \,\overline{x}$ follows from Corollary 5.2. It remains to show that $J_{\gamma A}T^{n}x_{0}\to J_{\gamma A}\overline{x}$ and $J_{\gamma B} R_{\gamma A}T^{n}x_{0}\to J_{\gamma B} R_{\gamma A} \overline{x}$. To this end, note that $(T^{n}x_{0})_{n\in{\mathbb{N}}}$ is bounded; consequently, since $J_{\gamma A}$ and $J_{\gamma B}R_{\gamma A}$ are Lipschitz continuous (see Proposition 2.16(i)&(ii)), we learn that

70 $$ \bigl(J_{\gamma A}T^{n}x_{0}\bigr)_{n\in{\mathbb{N}}} \text{ and } \bigl(J_{\gamma B}R_{\gamma A}T^{n}x_{0}\bigr)_{n\in{\mathbb{N}}}\text{ are bounded}. $$

On the one hand, in view of (68) we have

71 $$ (\operatorname{Id}-T)T^{n} x_{0}=T^{n} x_{0}-T^{n+1}x_{0} =J_{ \gamma A}T^{n}x_{0}-J_{\gamma B}R_{\gamma A}T^{n}x_{0} \to0. $$

Combining (70) and (71) yields

72a $$\begin{aligned} & \bigl\Vert J_{\gamma A}T^{n}x_{0}-J_{\gamma A} \overline{x} \bigr\Vert ^{2} - \bigl\Vert J_{\gamma B}R_{\gamma A}T^{n}x_{0} -J_{ \gamma B}R_{\gamma A}\overline{x} \bigr\Vert ^{2} \\ \end{aligned}$$

72b $$\begin{aligned} &\quad =\bigl\langle J_{\gamma A}T^{n}x_{0} -J_{\gamma B}R_{\gamma A}T^{n}x_{0} \mid J_{\gamma A}T^{n}x_{0} +J_{\gamma B}R_{\gamma A}T^{n}x_{0}-J_{ \gamma A} \overline{x} -J_{\gamma B}R_{\gamma A}\overline{x} \bigr\rangle \\ \end{aligned}$$

72c $$\begin{aligned} &\quad =\bigl\langle T^{n}x_{0}-T^{n+1}x_{0} \mid J_{\gamma A}T^{n}x_{0} +J_{ \gamma B}R_{\gamma A}T^{n}x_{0}-J_{\gamma A} \overline{x} -J_{\gamma B}R_{ \gamma A}\overline{x} \bigr\rangle \to0. \end{aligned}$$

On the other hand, combining Lemma 2.3, applied with $(R_{1},R_{2},R(\lambda),\lambda)$ replaced by $(R_{\gamma A},R_{\gamma B},T,1/2)$ and $(x,y) $ replaced by $(T^{n}x_{0}, \overline{x})$, in view of (68) yields

73a $$\begin{aligned} 0&\leftarrow\bigl\langle T^{n+1}x_{0}- \overline{x} \mid T^{n} x_{0}-T^{n+1}x_{0} \bigr\rangle \end{aligned}$$

73b $$\begin{aligned} &\ge\gamma\mu \biggl( \bigl\Vert J_{\gamma A}T^{n} x_{0}-J_{\gamma A} \overline{x} \bigr\Vert ^{2} -\frac{\omega}{\mu} \bigl\Vert J_{\gamma B}R_{ \gamma A}T^{n} x_{0}- J_{\gamma B}R_{\gamma A}\overline{x} \bigr\Vert ^{2} \biggr) \end{aligned}$$

73c $$\begin{aligned} &\ge - \frac{\gamma\mu\omega}{\mu-\omega} \bigl\Vert T^{n} x_{0}-T^{n+1}x_{0} \bigr\Vert ^{2}\to0. \end{aligned}$$

Therefore,

74 $$ \bigl\Vert J_{\gamma A}T^{n} x_{0}-J_{\gamma A}\overline{x} \bigr\Vert ^{2} - \frac{\omega}{\mu} \bigl\Vert J_{\gamma B} R_{\gamma A}T^{n} x_{0}-J_{ \gamma B}R_{\gamma A} \overline{x} \bigr\Vert ^{2}\to0. $$

Combining (72a)–(72c) and (74) and noting that $\frac{\omega}{\mu}<1$ yields $\Vert J_{\gamma A}T^{n} x_{0}-J_{\gamma A}\overline{x} \Vert ^{2}\to0$ and $\Vert J_{\gamma B}R_{\gamma A}T^{n} x_{0} -J_{\gamma B}R_{\gamma A} \overline{x} \Vert ^{2}\to0$, which proves (i). The proof of (ii) proceeds similarly. □

### Remark 5.6

(Relaxed Douglas–Rachford algorithm)

A careful look at the proofs of Theorem 5.1 and Theorem 5.5 reveals that analogous conclusions can be drawn for the relaxed Douglas–Rachford operator defined by $T_{\lambda}=(1-\lambda)\operatorname{Id}+\lambda R_{\gamma B}R_{ \gamma A} $, $\lambda\in\, ]0,1 [$. In this case, we choose $\gamma\in\, ]0, {((1-\lambda)(\mu-\omega))}/{(\mu\omega)} [$. One can verify that the corresponding averagedness constant is $\alpha =\frac{\lambda(\mu-\omega)}{\mu-\omega-\gamma\mu\omega} \in ]0,1 [$.

## Application to the forward–backward algorithm

Throughout this section we assume that

$$ \boxed{A\colon X\to X ,\qquad B\colon X\rightrightarrows X, \qquad \mu\ge0, \qquad \omega\ge0,\quad \text{and} \quad \beta>0.} $$

In the rest of this section, we prove that the forward–backward operator is averaged, hence its iterates form a weakly convergent sequence in each of the following situations:

- *A* is maximally *μ*-monotone, $A-\mu\operatorname{Id}$ is $\frac{1}{\beta}$-cocoercive, *B* is maximally $(-\omega)$-monotone, and $\mu\ge\omega$.
- *A* is maximally $(-\omega)$-monotone, $A+\omega\operatorname{Id}$ is $\frac{1}{\beta}$-cocoercive, *B* is maximally *μ*-monotone, and $\mu\ge\omega$.
- *A* is *β*-Lipschitz continuous, *B* is maximally *μ*-monotone, and $\mu\ge\beta$.

That is, we do not require *A* and *B* to be monotone. Instead, it is enough that the sum $A+B$ is monotone to have an averaged forward–backward map. In addition, we show that the forward–backward map is contractive if the sum $A+B$ is strongly monotone, and we prove the tightness of our contraction factor.

### Theorem 6.1

(Case I: *A* is *μ*-monotone)

*Let* $\mu\ge\omega\ge0$, *and let* $\beta>0$. *Suppose that* *A* *is maximally* *μ*-*monotone*, $A-\mu\operatorname{Id}$ *is* $\frac{1}{\beta}$-*cocoercive*, *and* *B* *is maximally* $(-\omega)$-*monotone*. *Let* $\gamma\in\, ]0, 2/(\beta+2\mu) [$. *Set* $T=J_{\gamma B}(\operatorname{Id}-\gamma A)$, *set* $\nu={\gamma\beta}/{(2(1-\gamma\mu))}$, *set* $\delta=(1-\gamma\mu)/(1-\gamma\omega)$, *and let* $x_{0}\in X$. *Then* $\delta\in\, ]0,1 ]$ *and* $\nu\in\, ]0,1 [ $. *Moreover*, *the following hold*:

(i) $T=\delta((1-\nu)\operatorname{Id}+\nu N)$, *N* *is nonexpansive*.
(ii) *T* *is* $(1-(\delta(1-\nu))/(2-\nu))$-*averaged*.
(iii) *T* *is* *δ*-*Lipschitz continuous*.
(iv) *There exists* $\overline{x}\in\operatorname{Fix}T =\operatorname{zer}(A+B) $ *such that* $T^{n}x_{0}\,\rightharpoonup \,\overline{x}$.

*Suppose that* $\mu>\omega$. *Then we additionally have*:

(v) *T* *is a Banach contraction with a constant* $\delta<1$.
(vi) $\operatorname{zer}(A+B)=\{\overline{x}\}$ *and* $T^{n}x_{0}\to\overline{x}$ *with a linear rate* $\delta<1$.

### Proof

Clearly, $\delta\in\, ]0,1 ] $ and $\nu>0$. Moreover, we have $\nu<1$ ⇔ $\gamma\beta<2(1-\gamma\mu)$ ⇔ $\gamma<2/(\beta+2\mu\beta)$. Hence, $\nu\in\, ]0,1 [ $ as claimed. Next note that $\mu<(\beta+2\mu)/2$, hence $\gamma\omega<\gamma\mu<(2\gamma)/(\beta+2\mu)<1$. It follows from Proposition 2.2 that $J_{\gamma B}$ and, in turn, *T* are single-valued and $\operatorname{dom}J_{\gamma B}=\operatorname{dom}T=X$. The assumption on *A* implies that there exists $\overline{N}\colon X\to X$, *N̅* is nonexpansive, such that $A-\mu\operatorname{Id}=\frac{\beta}{2 }\operatorname{Id}+ \frac{\beta}{2 }\overline{N}$. Therefore,

75a $$\begin{aligned} \operatorname{Id}-\gamma A &=\operatorname{Id}-\gamma( A-\mu \operatorname{Id})- \gamma\mu\operatorname{Id}=(1-\gamma\mu) \operatorname{Id}-\frac{\gamma\beta}{2}( \operatorname{Id}+ \overline{N}) \end{aligned}$$

75b $$\begin{aligned} &=(1-\gamma\mu) \bigl((1-\nu)\operatorname{Id}+\nu(-\overline{N}) \bigr). \end{aligned}$$

Moreover, Proposition 2.16(i) implies that

76 $$ \text{$J_{\gamma B}$ is $(1-\gamma\omega)$-cocoercive.} $$

(i): It follows from Corollary 4.3 applied with $(R_{1},R_{2})$ replaced by $(\operatorname{Id}-\gamma A,J_{\gamma B})$ and $(\alpha,\beta,\delta)$ replaced by $(\nu,1/(1-\gamma\omega),1-\gamma\mu)$, in view of (75a)–(75b) and (76), that there exists a nonexpansive operator *N* such that $T=J_{\gamma B}(\operatorname{Id}-\gamma A)=\delta((1-\nu) \operatorname{Id}+\nu N)$. (ii): Combine (i) and Lemma 2.15(i). (iii): Combine (i) and (ii). (iv): Applying Proposition 2.2 with $(A,B)$ replaced by $(\gamma A,\gamma B)$ yields $\operatorname{zer}(A+B)=\operatorname{zer}(\gamma A+\gamma B) = \operatorname{Fix}T$. The claim that $T^{n}x_{0}\,\rightharpoonup \,\overline{x}$ follows from combining (ii) and [2, Theorem 5.15]. (v): Observe that $\delta<1\Leftrightarrow\mu>\omega$. Now, combine with (iii). (vi): Note that $A+B$ is maximally $(\mu-\omega)$-monotone and $\mu-\omega>0$, we conclude from [2, Proposition 23.35] that $\operatorname{zer}(A+B)$ is a singleton. Alternatively, use (iii) to learn that *T* is a Banach contraction with a constant $\delta<1$, hence $\operatorname{zer}(A+B)=\operatorname{Fix}T$ is a singleton, and the conclusion follows. □

### Theorem 6.2

*Let* $\mu> \omega\ge0$, *and let* $\beta>0$. *Suppose that* *A* *is maximally* *μ*-*monotone*, $A-\mu\operatorname{Id}$ *is* $\frac{1}{\beta}$-*cocoercive*, *and* *B* *is maximally* $(-\omega)$-*monotone*. *Let* $\gamma\in [2/(\beta+2\mu), 2/(\beta+\mu) [$. *Set* $T=J_{\gamma B}(\operatorname{Id}-\gamma A)$, *set* $\nu={\gamma\beta}/{(2(\gamma(\mu+\beta)-1))}$, *set* $\delta=(1-\gamma(\mu+\beta))/(1-\gamma\omega)$, *and let* $x_{0}\in X$. *Then* $\delta\in\, ]{-}1,0 ]$ *and* $\nu\in\, ]0,1 [ $. *Moreover*, *the following hold*:

(i) $T=\delta((1-\nu)\operatorname{Id}+\nu N)$, *N* *is nonexpansive*.
(ii) *T* *is a Banach contraction with a constant* $|\delta|<1$.
(iii) *There exists* $\overline{x}\in X$ *such that* $\operatorname{Fix}T=\operatorname{zer}(A+B)=\{\overline{x}\}$ *and* $T^{n}x_{0}\to\overline{x}$ *with a linear rate* $\vert \delta \vert <1$.

### Proof

We proceed similar to the proof of Theorem 6.1 to verify that *T* is single-valued, $\operatorname{dom}T=X$, $\nu\in\, ]0,1 [$, and $\delta\in\, ]{-}1,0 ]$. The assumption on *A* implies that there exists $\overline{N}\colon X\to X$, *N̅* is nonexpansive such that $A-\mu\operatorname{Id}=\frac{\beta}{2 }\operatorname{Id}+ \frac{\beta}{2 }\overline{N}$. Therefore,

77a $$\begin{aligned} \operatorname{Id}-\gamma A &=\operatorname{Id}-\gamma( A-\mu \operatorname{Id})- \gamma\mu\operatorname{Id}=(1-\gamma\mu) \operatorname{Id}-\frac{\gamma\beta}{2}( \operatorname{Id}+ \overline{N}) \end{aligned}$$

77b $$\begin{aligned} &=\bigl(1-\gamma(\mu+\beta)\bigr) \bigl((1-\nu)\operatorname{Id}+\nu( \overline{N}) \bigr). \end{aligned}$$

Now, proceed similar to the proof of Theorem 6.1(i), (v), and (vi) in view of (76). □

### Corollary 6.3

*Let* $\mu> \omega\ge0$, *and let* $\beta>0$. *Suppose that* *A* *is maximally* *μ*-*monotone*, $A-\mu\operatorname{Id}$ *is* $\frac{1}{\beta}$-*cocoercive*, *and* *B* *is maximally* $(-\omega)$-*monotone*. *Let* $\gamma\in\, ]0, 2/(\beta+\mu) [$. *Set* $T=J_{\gamma B}(\operatorname{Id}-\gamma A)$, *set* $\delta=\max(1-\gamma\mu,\gamma(\mu+\beta)-1)/(1-\gamma \omega)$, *and let* $x_{0}\in X$. *Then* $\delta\in [0,1 [$, *T* *is a Banach contraction with a constant* *δ*, *and there exists* $\overline{x}\in X$ *such that* $\operatorname{Fix}T=\operatorname{zer}(A+B)=\{\overline{x}\}$ *and* $T^{n} x_{0}\to\overline{x}$.

### Proof

Combine Theorem 6.1 and Theorem 6.2. □

### Remark 6.4

(Tightness of the Lipschitz constant)

(i) Suppose that the setting of Theorem 6.1 holds. Set $(A,B)=(\mu\operatorname{Id},-\omega\operatorname{Id})$. Then $T=\frac{1-\gamma\mu}{1-\gamma\omega}\operatorname{Id}$. Hence, the claimed Lipschitz constant is tight.
(ii) Suppose that the setting of Theorem 6.2 holds. Set $(A,B)=((\mu+\beta)\operatorname{Id},-\omega\operatorname{Id})$. Then $T=\frac{\gamma(\mu+\beta)-1}{1-\gamma\omega}\operatorname{Id}$. Hence, the claimed contraction factor is tight.

Note in particular that the worst cases are subgradients of convex functions. Hence, the worst cases are attained by the proximal gradient method.

### Theorem 6.5

(Case II: $A+\omega\operatorname{Id}$ is cocoercive)

*Let* $\mu\ge\omega\ge0$, *let* $\beta>0$, *and let* $\overline{\beta}\in\, ]\max\{\beta, \mu+\omega\},+\infty [$. *Suppose that* *A* *is maximally* $(-\omega)$-*monotone*, $A+\omega\operatorname{Id}$ *is* *β*-*cocoercive*, *and* *B* *is maximally* *μ*-*monotone*. *Let* $\gamma\in\, ]0, 2/(\overline{\beta}-2\omega) [$. *Set* $T=J_{\gamma B}(\operatorname{Id}-\gamma A)$, *set* $\nu={\gamma\overline{\beta} }/{(2(1+\gamma\omega))}$, *set* $\delta=(1+\gamma\omega)/(1+\gamma\mu)$, *and let* $x_{0}\in X$. *Then* $\delta\in\, ]0,1 ]$ *and* $\nu\in\, ]0,1 [ $. *Moreover*, *the following hold*:

(i) $T=\delta((1-\nu)\operatorname{Id}+\nu N)$, *N* *is nonexpansive*.
(ii) *T* *is* $(1-(\delta(1-\nu))/(2-\nu))$-*averaged*.
(iii) *T* *is* *δ*-*Lipschitz continuous*.
(iv) *There exists* $\overline{x}\in\operatorname{Fix}T =\operatorname{zer}(A+B) $, *and* $T^{n}x_{0}\,\rightharpoonup \,\overline{x}$.

*Suppose that* $\mu>\omega$. *Then we additionally have*:

(v) *T* *is a Banach contraction with a constant* $\delta<1$.
(vi) $\operatorname{zer}(A+B)=\{\overline{x}\}$ *and* $T^{n}x_{0}\to\overline{x}$ *with a linear rate* $\delta<1$.

### Proof

Observe that the assumption on *A* and Lemma 2.11 applied with *T* replaced by $A+\omega\operatorname{Id}$ imply that there exists $\overline{N}\colon X\to X$, *N̅* is nonexpansive, such that $A+\omega\operatorname{Id}=\frac{\overline{\beta}}{2 } \operatorname{Id}+\frac{\overline{\beta}}{2}\overline{N}$.

78a $$\begin{aligned} \operatorname{Id}-\gamma A &=\operatorname{Id}-\gamma( A+\omega \operatorname{Id})+\gamma\omega\operatorname{Id}=(1+\gamma \omega) \operatorname{Id}-\frac{\gamma\overline{\beta}}{2}( \operatorname{Id}+\overline{N}) \end{aligned}$$

78b $$\begin{aligned} &=(1+\gamma\omega) \bigl((1-\nu)\operatorname{Id}+\nu(- \overline{N}) \bigr). \end{aligned}$$

Moreover, Proposition 2.16(i) implies that

79 $$ \text{$J_{\gamma B}$ is $(1+\gamma\mu)$-cocoercive.} $$

Now proceed similar to the proof of Theorem 6.1 but use (78a)–(78b) and (79). □

### Theorem 6.6

*Let* $\mu> \omega\ge0$, *let* $\beta>0$, *and let* $\overline{\beta}\in\, ]\max\{\beta, \mu+\omega\},+\infty [$. *Suppose that* *A* *is maximally* $(-\omega)$-*monotone*, $A+\omega\operatorname{Id}$ *is* *β*-*cocoercive*, *and* *B* *is maximally* *μ*-*monotone*. *Let* $\gamma\in [ 2/(\overline{\beta}-2\omega),2/(\overline{\beta}- \mu-\omega) [$. *Set* $T=J_{\gamma B}(\operatorname{Id}-\gamma A)$, *set* $\nu={\gamma\overline{\beta} }/{(2(\gamma\overline{\beta}-\gamma \omega-1))}$, *set* $\delta=(1+\gamma\omega-\gamma\overline{\beta})/(1+\gamma\mu)$, *and let* $x_{0}\in X$. *Then* $\delta\in\, ]{-}1,0 ]$ *and* $\nu\in\, ]0,1 [ $. *Moreover*, *the following hold*:

(i) $T=\delta((1-\nu)\operatorname{Id}+\nu N)$, *N* *is nonexpansive*.
(ii) *T* *is a Banach contraction with a constant* $\vert \delta \vert <1$.
(iii) *There exists* $\overline{x}\in X$ *such that* $\operatorname{Fix}T=\operatorname{zer}(A+B)=\{\overline{x}\}$ *and* $T^{n}x_{0}\to\overline{x}$ *with a linear rate* $\vert \delta \vert <1$.

### Proof

Observe that the assumption on *A* and Lemma 2.11 applied with *T* replaced by $A+\omega\operatorname{Id}$ implies that there exists $\overline{N}\colon X\to X$, *N̅* is nonexpansive, such that $A+\omega\operatorname{Id}=\frac{\overline{\beta}}{2 } \operatorname{Id}+\frac{\overline{\beta}}{2}\overline{N}$.

80a $$\begin{aligned} \operatorname{Id}-\gamma A &=\operatorname{Id}-\gamma( A+\omega \operatorname{Id})+\gamma\omega\operatorname{Id}=(1+\gamma \omega) \operatorname{Id}-\frac{\gamma\overline{\beta}}{2}( \operatorname{Id}+\overline{N}) \end{aligned}$$

80b $$\begin{aligned} &=(1+\gamma\omega-\gamma\overline{\beta}) \bigl((1-\nu) \operatorname{Id}+\nu \overline{N} \bigr). \end{aligned}$$

Now proceed similar to the proof of Theorem 6.5 in view of (79). □

### Corollary 6.7

*Let* $\mu> \omega\ge0$, *let* $\beta>0$, *and let* $\overline{\beta}\in\, ]\max\{\beta, \mu+\omega\},+\infty [$. *Suppose that* *A* *is maximally* $(-\omega)$-*monotone*, $A+\omega\operatorname{Id}$ *is* *β*-*cocoercive*, *and* *B* *is maximally* *μ*-*monotone*. *Let* $\gamma\in [ 0,2/(\overline{\beta}-\mu-\omega) [$. *Set* $T=J_{\gamma B}(\operatorname{Id}-\gamma A)$, *set* $\delta=\max\{1+\gamma\mu,\gamma\overline{\beta}-\gamma\omega-1 \}/(1+\gamma\mu)$, *and let* $x_{0}\in X$. *Then* $\delta\in\, ]{-}1,0 ]$ *and* $\nu\in\, ]0,1 [ $. *Then* $\delta\in [0,1 [$, *T* *is a Banach contraction with a constant* *δ*, *and there exists* $\overline{x}\in X$ *such that* $\operatorname{Fix}T=\operatorname{zer}(A+B)=\{\overline{x}\}$ *and* $T^{n} x_{0}\to\overline{x}$.

### Proof

Combine Theorem 6.5 and Theorem 6.6. □

### Theorem 6.8

(Case III: *A* is *β*-Lipschitz continuous)

*Let* $\mu\ge\beta>0$. *Suppose that* *A* *is* *β*-*Lipschitz continuous and that* *B* *is maximally* *μ*-*monotone*. *Let* $\overline{\beta}\in\, ]2\beta,+\infty [$, *and let* $\gamma\in\, ]0,2 /( \overline{\beta}-2\beta)\} [$. *Set* $T=J_{\gamma B}(\operatorname{Id}-\gamma A)$, *set* $\nu={\gamma\overline{\beta} }/{(2(1+\gamma\beta))}$, *set* $\delta=(1+\gamma\beta)/(1+\gamma\mu)$, *and let* $x_{0}\in X$. *Then* $\delta\in\, ]0,1 ]$ *and* $\nu\in\, ]0,1 [ $. *Moreover*, *the following hold*:

(i) $T=\delta((1-\nu)\operatorname{Id}+\nu N)$, *N* *is nonexpansive*.
(ii) *T* *is* $(1-(\delta(1-\nu))/(2-\nu))$-*averaged*.
(iii) *T* *is* *δ*-*Lipschitz continuous*.
(iv) *There exists* $\overline{x}\in\operatorname{Fix}T =\operatorname{zer}(A+B) $, *and* $T^{n}x_{0}\,\rightharpoonup \,\overline{x}$.

*Suppose that* $\mu>1/\beta$. *Then we additionally have*:

(v) *T* *is a Banach contraction with a constant* $\delta<1$.
(vi) $\operatorname{zer}(A+B)=\{\overline{x}\}$ *and* $T^{n}x_{0}\to\overline{x}$ *with a linear rate* $\delta<1$.

### Proof

Combine Lemma 2.12 and Theorem 6.5 applied with $(\omega,\beta)$ replaced by $(\beta,2\beta)$. □

### Theorem 6.9

*Let* $\mu> \beta> 0$. *Suppose that* *A* *is* *β*-*Lipschitz continuous and that* *B* *is maximally* *μ*-*monotone*. *Let* $\overline{\beta}\in\, ]\mu+\beta,+\infty [$, *and let* $\gamma\in [ 2/(\overline{\beta}-2\beta),2/(\overline{\beta}- \mu-\beta) [$. *Set* $T=J_{\gamma B}(\operatorname{Id}-\gamma A)$, *set* $\nu={\gamma\overline{\beta} }/{(2(\gamma\overline{\beta}-\gamma \beta-1))}$, *set* $\delta=(1+\gamma\beta-\gamma\overline{\beta})/(1+\gamma\mu)$, *and let* $x_{0}\in X$. *Then* $\delta\in\, ]{-}1,0 ]$ *and* $\nu\in\, ]0,1 [ $. *Moreover*, *the following hold*:

(i) $T=\delta((1-\nu)\operatorname{Id}+\nu N)$, *N* *is nonexpansive*.
(ii) *T* *is a Banach contraction with a constant* $\vert \delta \vert <1$.
(iii) *There exists* $\overline{x}\in X$ *such that* $\operatorname{Fix}T=\operatorname{zer}(A+B)=\{\overline{x}\}$ *and* $T^{n}x_{0}\to\overline{x}$ *with a linear rate* $\vert \delta \vert <1$.

### Proof

Combine Lemma 2.12 and Theorem 6.6 applied with $(\omega,\beta)$ replaced by $(\beta,2\beta)$. □

## Applications to optimization problems

Let $f\colon X\to ]{-}\infty,+\infty ]$, and let $g\colon X\to ]{-}\infty,+\infty ]$. Throughout this section, we shall assume that

$$ \boxed{\text{$f$ and $g$ are proper lower semicontinuous functions.}} $$

We shall use *∂f* to denote the subdifferential mapping from convex analysis.

### Definition 7.1

(see [3, Definition 6.1])

An abstract subdifferential $\partial_{\text{\#}}$ associates a subset $\partial_{\text{\#}}f(x)$ of *X* with *f* at $x\in X$, and it satisfies the following properties:

(i) $\partial_{\text{\#}}f=\partial f$ if *f* is a proper lower semicontinuous convex function;
(ii) $\partial_{\text{\#}}f=\nabla f$ if *f* is continuously differentiable;
(iii) $0\in\partial_{\text{\#}}f(x)$ if *f* attains a local minimum at $x\in\operatorname{dom}f$;
(iv) for every $\beta\in{\mathbb{R}}$,
  $$ \partial_{\text{\#}} \biggl(f+\beta\frac{ \Vert \cdot-x \Vert ^{2}}{2} \biggr)= \partial_{\text{\#}}f +\beta(\operatorname{Id}-x). $$

The Clarke–Rockafellar subdifferential, Mordukhovich subdifferential, and Frechét subdifferential all satisfy Definition 7.1(i)–(iv), see, e.g., [5, 19, 20], so they are $\partial_{\text{\#}}$.

Let $\lambda>0$. Recall that *f* is *λ*-hypoconvex (see [23, 26]) if

81 $$ f\bigl((1-\tau)x+\tau y\bigr)\le(1-\tau) f(x) +\tau f(y) + \frac{\lambda}{2} \tau(1-\tau) \Vert x-y \Vert ^{2} $$

for all $(x,y)\in X\times X$ and $\tau\in\, ]0,1 [$ or, equivalently,

82 $$ \text{$f+\frac{\lambda}{2} \Vert \cdot \Vert ^{2}$ is convex.} $$

For $\gamma>0$, the *proximal mapping* $\mathop{\operatorname{Prox}_{\gamma f}}$ is defined at $x\in X$ by

83 $$ \mathop{\operatorname{Prox}_{\gamma f}}(x) = \underset{y\in X}{\operatorname*{argmin}} \biggl(f(y)+\frac{\gamma}{2} \Vert x-y \Vert ^{2} \biggr). $$

### Fact 7.2

*Suppose that* $f\colon X\to ]{-}\infty,+\infty ]$ *is a proper lower semicontinuous* *λ*-*hypoconvex function*. *Then*

84 $$ \partial_{\textit{\#}}f=\partial \biggl(f+ \frac{\lambda}{2} \Vert \cdot \Vert ^{2} \biggr)-{\lambda} \operatorname{Id}. $$

*Moreover*, *we have*:

(i) *The Clarke–Rockafellar*, *Mordukhovich*, *and Frechét subdifferential operators of* *f* *all coincide*.
(ii) $\partial_{\textit{\#}}f$ *is maximally* −*λ*-*monotone*.
(iii) $(\forall\gamma\in\, ]0,{1}/{\lambda} [)$ $\mathop{\operatorname{Prox}_{\gamma f}}$ *is single*-*valued and* $\operatorname{dom}\mathop{\operatorname{Prox}_{\gamma f}}=X$.

### Proof

See [3, Proposition 6.2 and Proposition 6.3]. □

### Proposition 7.3

*Let* $\mu\ge\omega\ge0$. *Suppose that* $\operatorname*{argmin}(f+g)\neq\varnothing$ *and that one of the following conditions is satisfied*:

(i) *f* *is* *μ*- *strongly convex*, *g* *is* *ω*- *hypoconvex*.
(ii) *f* *is* *ω*- *hypoconvex*, *and* *g* *is* *μ*- *strongly convex*.

*Then* $f+g$ *is convex and* $\partial_{\textit{\#}}(f+g)=\partial(f+g)$.

*If*, *in addition*, *one of the following conditions is satisfied*:

- $0\in\operatorname{sri}(\operatorname{dom}f-\operatorname{dom}g)$.
- *X* *is finite dimensional and* $0\in\operatorname{ri}(\operatorname{dom}f-\operatorname{dom}g)$.
- *X* *is finite dimensional*, *f* *and* *g* *are polyhedral*, *and* $\operatorname{dom}f\cap\operatorname{dom}g\neq\varnothing$.

*Then*

85 $$ \partial_{\textit{\#}}(f+g)=\partial(f+g)= \partial_{\textit{\#}}f+ \partial_{\textit{\#}}g, $$

*and*

86 $$ \operatorname{zer}\partial_{\textit{\#}}(f+g)= \operatorname{zer}( \partial_{\textit{\#}}f+\partial_{\textit{\#}}g)= \operatorname*{argmin}(f+g). $$

### Proof

It is clear that either (i) or (ii) implies that $f+g$ is convex, and the identity follows in view of Definition 7.1(i). Now, suppose that (i) holds along with one of the assumptions (a)–(c). Rewrite *f* and *g* as $(f,g)=(\overline{f}+\frac{\mu}{2} \Vert \cdot \Vert ^{2}, \overline{g}-\frac{\omega}{2} \Vert \cdot \Vert ^{2})$ and observe that both *f̅* and *g̅* are convex, as is $\overline{f}+\overline{g}$. Moreover, we have $\operatorname{dom}f=\operatorname{dom}\overline{f}$ and $\operatorname{dom}g=\operatorname{dom}\overline{g}$. Now,

87a $$\begin{aligned} \partial_{\text{\#}}(f+g) &=\partial_{\text{\#}}\biggl(\overline{f}+ \overline{g} +\frac{\mu-\omega}{2} \Vert \cdot \Vert ^{2}\biggr) \end{aligned}$$

87b $$\begin{aligned} &=\partial_{\text{\#}}(\overline{f}+\overline{g}) +({\mu-\omega}) \operatorname{Id}=\partial(\overline{f}+\overline{g}) +({\mu- \omega}) \operatorname{Id} \end{aligned}$$

87c $$\begin{aligned} &=\partial\overline{f}+\partial\overline{g} +({\mu-\omega}) \operatorname{Id}= \partial\overline{f}+\mu\operatorname{Id}+ \partial\overline{g}-\omega \operatorname{Id} \end{aligned}$$

87d $$\begin{aligned} &=\partial f+\partial_{\text{\#}}g=\partial_{\text{\#}}f+ \partial_{ \text{\#}}g. \end{aligned}$$

Here, (87b) follows from applying Definition 7.1(iv) to $\overline{f}+\overline{g}$, (87c) follows from [2, Theorem 16.47] applied to *f̅* and *g̅*, and (87c) follows from applying Fact 7.2 to *f* and *g* and using Definition 7.1(i), which verify (85). Finally, (86) follows from combining (85) and [2, Theorem 16.3]. □

The following theorem provides an alternative proof to [17, Theorem 4.4] and [9, Theorem 5.4(ii)].

### Theorem 7.4

*Let* $\mu>\omega\ge0$, *and let* $\gamma\in\, ]0, {(\mu-\omega)}/(2\mu\omega) [$. *Suppose that one of the following holds*:

(i) *f* *is* *μ*- *strongly convex*, *g* *is* *ω*- *hypoconvex*.
(ii) *f* *is* *ω*-*hypoconvex*, *and* *g* *is* *μ*-*strongly convex*,

*and that* $0\in\partial_{\textit{\#}}f+\partial_{\textit{\#}}g$ (*see Proposition *7.3*for sufficient conditions*). *Set*

88 $$ T=\frac{1}{2} \bigl(\operatorname{Id}+ (2\mathop{ \operatorname{Prox}_{\gamma g}}-\operatorname{Id}) (2\mathop{ \operatorname{Prox}_{\gamma f}}-\operatorname{Id}) \bigr) \quad \textit{and}\quad \alpha= \frac{\mu-\omega}{2(\mu-\omega-\gamma\mu\omega)}, $$

*and let* $x_{0}\in X$. *Then* $\alpha\in\, ]0,1 [$, *and* *T* *is* *α*-*averaged*. *Moreover*, $(\exists \overline{x}\in\operatorname{Fix}T)$ *such that* $T^{n} x_{0}\,\rightharpoonup \,\overline{x}$, $\operatorname*{argmin}(f+g)=\{ \mathop{\operatorname{Prox}_{f}} \overline{x}\}$, *and* $\mathop{\operatorname{Prox}_{f}}T^{n} x_{0} \to\mathop{ \operatorname{Prox}_{f}} \overline{x}$.

### Proof

Suppose that (i) holds. Then [2, Example 22.4] (respectively Fact 7.2(ii)) implies that $\partial_{\text{\#}}f=\partial f$ (respectively $\partial_{\text{\#}}g$) is maximally *μ*-monotone (respectively maximally $(-\omega)$-monotone). The conclusion follows from applying Theorem 5.5(i) with $(A,B)$ replaced by $(\partial_{\text{\#}}f,\partial_{\text{\#}}g)$. The proof for (ii) follows similarly by using Theorem 5.5(ii). □

Before we proceed further, we recall the following useful fact.

### Fact 7.5

(Baillon–Haddad)

*Let* $f\colon X\to{\mathbb{R}}$ *be a Frechét differentiable convex function*, *and let* $\beta>0$. *Then* $\mathop{\nabla{f} } $ *is* *β*-*Lipschitz continuous if and only if* $\mathop{\nabla{f} } $ *is* $\frac{1}{\beta}$-*cocoercive*.

### Proof

See, e.g., [2, Corollary 18.17]. □

### Lemma 7.6

*Let* $\mu\ge0$, *let* $\beta>0$, *and let* $f\colon X\to{\mathbb{R}}$ *be a Frechét differentiable function*. *Suppose that* *f* *is* *μ*-*strongly convex with a* *β*-*Lipschitz continuous gradient*. *Then the following hold*:

(i) $f-\frac{\mu}{2} \Vert \cdot \Vert ^{2}$ *is convex*.
(ii) $\mathop{\nabla{f} } $ *is maximally* *μ*-*monotone*.
(iii) $\mathop{\nabla{f} } -\mu\operatorname{Id}$ *is* $\frac{1}{\beta}$-*cocoercive*.

### Proof

(i): See, e.g., [2, Proposition 10.8]. (ii): See, e.g., [2, Example 22.4(iv)]. (iii): Combine (i), Lemma 2.10, and Corollary 2.14(ii) applied with $(f_{1},f_{2})$ replaced by $(f, \frac{\mu}{2} \Vert \cdot \Vert ^{2})$. □

### Theorem 7.7

(The forward–backward algorithm when *f* is *μ*-strongly convex)

*Let* $\mu\ge\omega\ge0$, *and let* $\beta>0$. *Let* *f* *be* *μ*-*strongly convex and Frechét differentiable with a* *β*-*Lipschitz continuous gradient*, *and let* *g* *be* *ω*-*hypoconvex*. *Suppose that* $\operatorname*{argmin}(f+g)\neq\varnothing$. *Let* $\gamma\in\, ]0,2/(\beta+2\mu) [$, *and set* $\delta=(1-\gamma\mu)/(1-\gamma\omega)$. *Set* $T=\operatorname{Prox}_{\gamma g}(\operatorname{Id}-\gamma\mathop {\nabla{f} } )$, *and let* $x_{0}\in X$. *Then the following hold*:

(i) *There exists* $\overline{x}\in\operatorname{Fix}T=\operatorname{zer}(A+B)= \operatorname*{argmin}(f+g)$ *such that* $T^{n} x_{0}\,\rightharpoonup \,\overline{x}$.

*Suppose that* $\mu>\omega$. *Then we additionally have*:

(ii) $\operatorname{Fix}T=\operatorname*{argmin}(f+g)=\{ \overline{x}\}$ *and* $T^{n} x_{0}\to\overline{x}$ *with a linear rate* $\delta<1$.

### Proof

Note that Definition 7.1(ii) implies that $\partial_{\text{\#}}f=\mathop{\nabla{f} } $. Set $(A,B)=(\mathop{\nabla{f} } ,\partial_{\text{\#}}g)$ and observe that Proposition 7.3 and Proposition 2.2 imply that $\operatorname{Fix}T=\operatorname{zer}(A+B)=\operatorname*{argmin}(f+g)$. It follows from [2, Example 22.4] (respectively Fact 7.2(ii)) that *A* (respectively *B*) is maximally *μ*-monotone (respectively maximally $(-\omega)$-monotone). Moreover, Lemma 7.6(iii) implies that $A-\mu\operatorname{Id}$ is $\frac{1}{\beta}$-cocoercive. (i)–(ii): Apply Theorem 6.1(iv)&(vi). □

To proceed to the next result, we need the following lemma.

### Lemma 7.8

*Let* $\omega\ge0$, *let* $\beta>0$, *and let* $f\colon X\to{\mathbb{R}}$ *be a Frechét differentiable function*. *Suppose that* *g* *is* *ω*-*hypoconvex with a* $\frac{1}{\beta}$-*Lipschitz continuous gradient*. *Then* $\mathop{\nabla{f} } +\omega\operatorname{Id}$ *is* $\beta/(1+\omega\beta)$-*cocoercive*.

### Theorem 7.9

(The forward–backward algorithm when *f* is *ω*-hypoconvex)

*Let* $\mu\ge\omega\ge0$, *let* $\beta>0$, *and let* $\overline{\beta}\in\, ] \max\{\beta,2\omega\},+\infty [$. *Let* *f* *be* *ω*-*hypoconvex*, *and let* *g* *be* *μ*-*strongly convex and Frechét differentiable with a* *β*-*Lipschitz continuous gradient*. *Suppose that* $\operatorname*{argmin}(f+g)\neq\varnothing$. *Let* $\gamma\in\, ]0, 2/(\overline{\beta}-2\omega) [$, *and set* $\delta=(1+\gamma\omega)/(1+\gamma\mu)$. *Set* $T=\operatorname{Prox}_{\gamma g}(\operatorname{Id}-\gamma\mathop {\nabla{f} } )$, *and let* $x_{0}\in X$. *Then the following hold*:

(i) *There exists* $\overline{x}\in\operatorname{Fix}T=\operatorname*{argmin}(f+g)$ *such that* $T^{n} x_{0}\,\rightharpoonup \,\overline{x}$.

*Suppose that* $\mu>\omega$. *Then we additionally have*:

(ii) $\operatorname{Fix}T=\operatorname*{argmin}(f+g)=\{ \overline {x}\}$ *and* $T^{n} x_{0}\to\overline{x}$ *with a linear rate* $\delta<1$.

### Proof

Proceed similar to the proof of Theorem 7.7 but use Theorem 6.5(iv)&(vi). □

### Theorem 7.10

(The forward–backward algorithm when *f* is $1/\beta$-hypoconvex)

*Let* $\mu\ge\beta>0$, *and let* $\overline{\beta}\in\, ]2\beta,+\infty [$. *Let* *f* *be* *μ*-*strongly convex*, *and let* *g* *be Frechét differentiable with a* *β*-*Lipschitz continuous gradient*. *Suppose that* $\operatorname*{argmin}(f+g)\neq\varnothing$. *Let* $\gamma\in\, ]0,2 /( \overline{\beta}-2\beta)\} [$, *and set* $\delta=(1+\gamma\beta)/(1+\gamma\mu)$. *Set* $T=\operatorname{Prox}_{\gamma g}(\operatorname{Id}-\gamma\mathop {\nabla{f} } )$, *and let* $x_{0}\in X$. *Then the following hold*:

(i) *There exists* $\overline{x}\in\operatorname{Fix}T=\operatorname*{argmin}(f+g)$ *such that* $T^{n} x_{0}\,\rightharpoonup \,\overline{x}$.

*Suppose that* $\mu>1/\beta$. *Then we additionally have*:

(ii) $\operatorname{Fix}T=\operatorname*{argmin}(f+g)=\{ \overline {x}\}$ *and* $T^{n} x_{0}\to\overline{x}$ *with a linear rate* $\delta<1$.

### Proof

Combine Lemma 2.12 applied with *A* replaced by $\mathop{\nabla{f} } $ and Theorem 7.9 applied with $(\omega,\beta)$ replaced by $(\beta,2\beta)$. □

### Remark 7.11

The results of Theorem 6.2, Theorem 6.6, and Theorem 6.9 can be directly applied to optimization settings in a similar fashion à la Theorem 7.7, Theorem 7.9, and Theorem 7.10.

## Graphical characterizations

This section contains 2D-graphical representations of different Lipschitz continuous operator classes that admit I-N decompositions and of their composition classes. We illustrate exact shapes of the composition classes in 2D and conservative estimates from Theorem 3.4 and Theorem 4.2. Similar graphical representations have appeared before in the literature. In [10, 11], nonexpansiveness and firm nonexpansiveness ($\frac{1}{2}$-averagedness) are characterized. Early preprints of [15] have more 2D graphical representations, and the lecture notes [14] contain many such characterizations with the purpose of illustrating how different properties relate to each other and to provide intuition on why different algorithms converge. This has been further extended and formalized in [24]. Not only do these illustrations provide intuition. Indeed, it is a straightforward consequence of, e.g., [24, 25] that for compositions of two operator classes that admit I-N decompositions, there always exists a 2D-worst case. Hence, if the 2D illustration implies that the composition class admits a specific $(\alpha,\beta)$-I-N decomposition, so does the full operator class.

In Sect. 8.1, we characterize many well-known special cases of operator classes that admit I-N decompositions. In Sect. 8.2, we characterize classes obtained by compositions of such operator classes and highlight differences between the true composition classes and their characterizations using Theorem 3.4.

### Single operators

We consider classes of $(\alpha,\beta)$-I-N decomposition of Lipschitz continuous operators. We graphically illustrate properties of some special cases. The illustrations should be read as follows. Assume that $x-y$ is represented by the marker in the figure. The diagram then shows where $Rx-Ry$ can end up in relation to $x-y$. If the point $x-y$ is rotated in the picture, the rest of the picture rotates with it. The characterization is, by construction of $(\alpha,\beta)$-I-N decompositions, always a circle of radius $\beta\|x-y\|$ shifted $\alpha\|x-y\|$ along the line defined by the origin and the point $x-y$.

#### Lipschitz continuous operators

**Figure 1:**
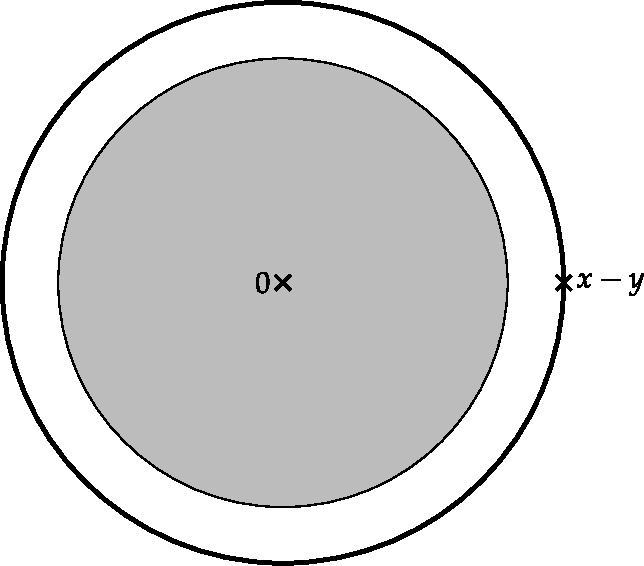
Illustration of *β*-Lipschitz continuous operator with $\beta=0.8$

Let $\beta>0$ and let $R\colon X\to X$. Then *R* is *β*-Lipschitz continuous if and only if *R* admits an $(\alpha,\beta)$-I-N decomposition, with *α* chosen as 0. Figure 1 shows the case $\beta=0.8 $. The radius of the Lipschitz circle is $\beta\|x-y\|$.

#### Cocoercive operators

**Figure 2:**
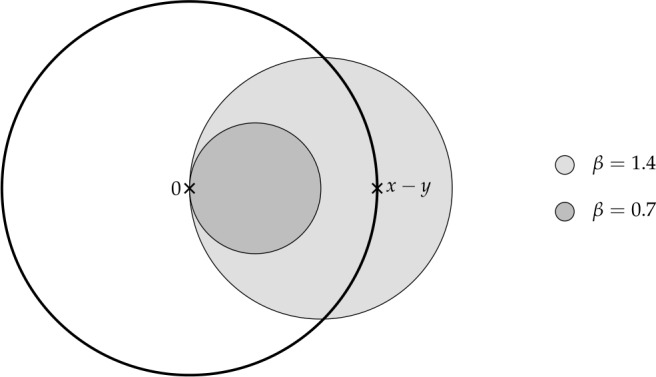
Illustration of $\tfrac{1}{\beta}$-cocoercive operators with $\beta=0.7$ and $\beta=1.4$

Let $\beta>0$, and let $R\colon X\to X$. Then *R* is $\frac{1}{\beta}$-cocoercive if and only if *R* admits an $(\alpha,\beta)$-I-N decomposition, with $(\alpha,\beta)$ chosen as $(\frac{\beta}{2},\frac{\beta}{2} )$. Figure 2 shows the cases $\beta=1.4 $ and $\beta=0.7 $. The diameter is $\beta\|x-y\|$. The figure clearly illustrates that $\frac{1}{\beta}$-cocoercive operators are also *β*-Lipschitz (but not necessarily the other way around).

#### Averaged operators

**Figure 3:**
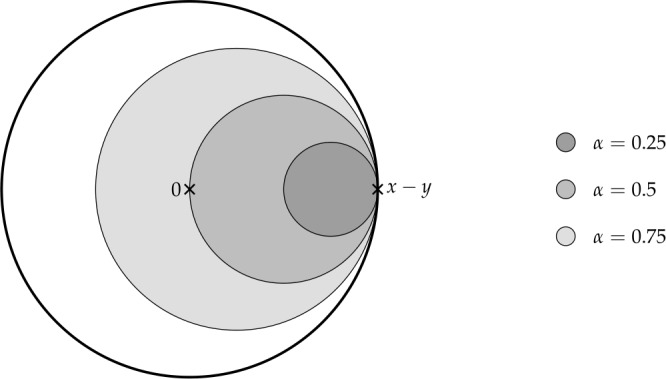
Illustration of *α*-averaged operators with $\alpha=0.25$, $\alpha=0.5$, and $\alpha=0.75$

Let $\alpha\in\, ]0,1 [$, and let $R\colon X\to X$. Then *R* is *α*-averaged if and only if *R* admits an $(\alpha,\beta)$-I-N decomposition, with $(\alpha,\beta)$ chosen as $(1-\alpha,\alpha )$. Figure 3 shows the cases $\alpha=0.25$ and $\alpha=0.5$, and $\alpha=0.75$. All averaged operators are nonexpansive.

#### Conic operators

**Figure 4:**
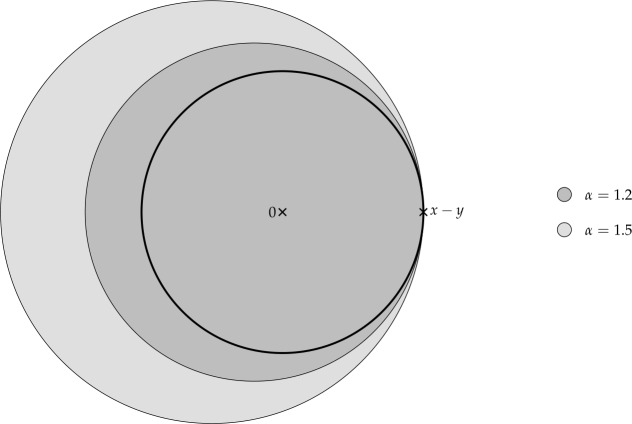
Illustration of *α*-conically nonexpansive operators with $\alpha=1.2$ and $\alpha=1.5$

Let $\alpha>0$, and let $R\colon X\to X$. Then *R* is *α*-conically nonexpansive if and only if *R* admits an $(\alpha,\beta)$-I-N decomposition, with $(\alpha,\beta)$ chosen as $(1-\alpha,\alpha )$. Figure 4 shows the cases $\alpha=1.2$ and $\alpha=1.5$. Conically nonexpansive operators fail to be nonexpansive for $\alpha>1$.

#### *μ*-Monotone operators

**Figure 5:**
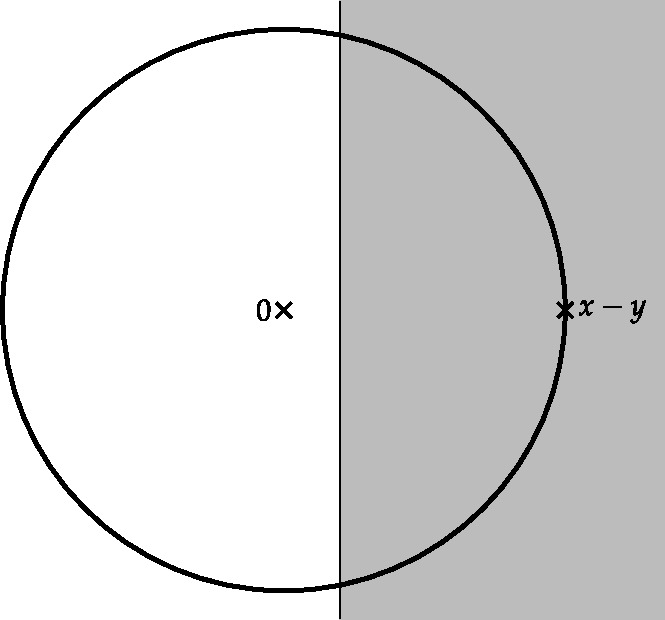
Illustration of *μ*-monotone operator with $\mu=0.2$

Let $\mu\in{\mathbb{R}}$, and suppose that $A\colon X\rightrightarrows X$ is *μ*-monotone. The shortest distance between the vertical line and the origin in the illustration is $|\mu|\|x-y\|$. Figure 5 shows the case $\mu=0.2$.

### Compositions of two operators

In this section, we provide illustrations of compositions of different classes of Lipschitz continuous operators. We consider compositions of the form

$$ R=R_{2}R_{1}, \quad \text{where $R_{i}$ admits an $(\alpha_{i},\beta_{i})$-I-N decomposition}, $$

$\forall i\in\{1,2\}$. Let $(x,y)\in X\times X$. We illustrate the regions within which $R_{2}R_{1}x-R_{2}R_{1}y$ can end up. For most considered composition classes, we provide two illustrations. The left illustration explicitly shows how the composition is constructed. It shows the region within which $R_{1}x-R_{1}y$ must end up. The second operator $R_{2}$ is applied at a subset, marked by crosses, of boundary points of that region. Given these as starting points for $R_{2}$ application, the dashed circles show where $R_{2}R_{1}x-R_{2}R_{1}y$ can end up for this subset. The right illustration shows, in gray, the resulting exact shape of the composition. It also contains the estimate from Theorem 3.4 that provides an I-N decomposition of the composition. From these illustrations, it is obvious that many different I-N decomposition are valid. The illustrations also reveal that the specific I-N decompositions provided in Theorem 3.4 indeed are suitable for our purpose of characterizing the composition as averaged, conic, or contractive.

#### Averaged-averaged composition

**Figure 6:**
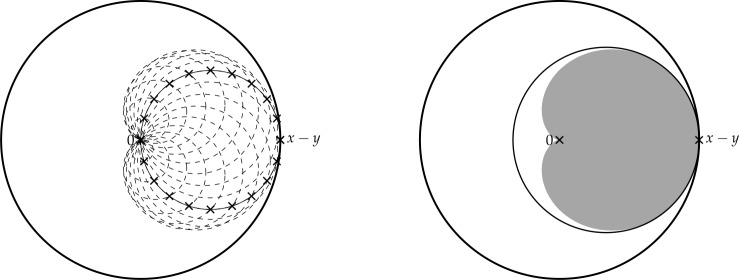
Illustration of composition of $\alpha_{1}$-averaged and $\alpha_{2}$-averaged operators with $\alpha_{1}=\alpha_{2}=0.5$

We first consider $\alpha_{i}$-averaged $R_{i}$ with $\alpha_{i}\in]0,1[$. A special case is the forward–backward splitting operator $T=J_{\gamma B}({\mathrm{Id}}-\gamma A)$ with $\frac {1}{\beta}$-cocoercive *A* and maximally monotone *B*. This implies that $({\mathrm{Id}}-\gamma A)$ is $\frac{\gamma\beta}{2}$-averaged for $\gamma\in\, ]0,\frac{2}{\beta} [$ and that $J_{\gamma B}$ is $\frac{1}{2}$-averaged. The example in Fig. 6 has individual averagedness parameters $\alpha_{1}=0.5 $ and $\alpha_{2}=0.5 $, i.e., $R=R_{2}R_{1}$ with $R_{1}=0.5 {\mathrm{Id}}+0.5 N_{1}$ and $R_{2}=0.5 {\mathrm{Id}}+0.5 N_{2}$. Theorem 3.4 shows that the composition is of the form $0.33 {\mathrm{Id}}+ 0.67 N$, where *N* is nonexpansive, i.e., it is 0.67-averaged. The fact that the composition is averaged is already known, see [8, 12].

**Figure 7:**
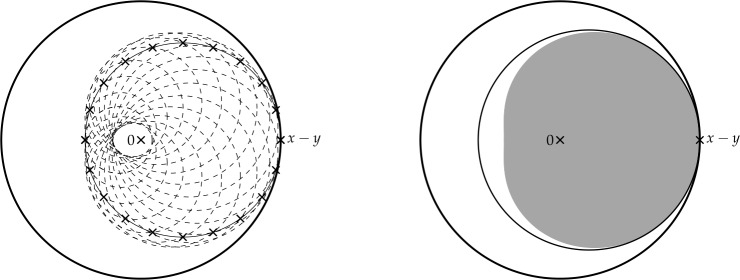
Illustration of composition of $\alpha_{1}$-averaged and $\alpha_{2}$-averaged operators with $\alpha_{1}=0.7$ and $\alpha_{2}=0.6$

The example in Fig. 7 shows $\alpha_{1}=0.7 $ and $\alpha_{2}=0.6 $. Theorem 3.4 shows that the composition is of the form $0.21 {\mathrm{Id}}+ 0.79 N$, where *N* is nonexpansive, i.e., it is 0.79-averaged.

#### Conic-conic composition

**Figure 8:**
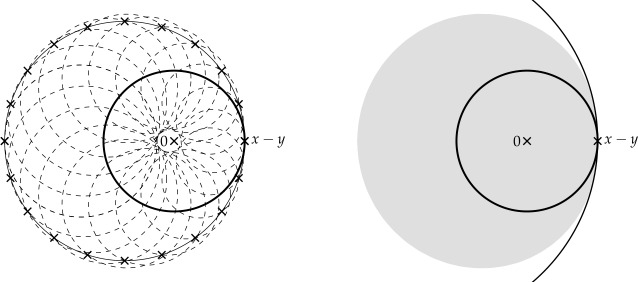
Illustration of composition of $\alpha_{1}$-conic operator and $\alpha_{2}$-averaged operator with $\alpha_{1}=1.7$ and $\alpha_{2}=0.45$

We consider $\alpha_{i}$-averaged $R_{i}$ with $\alpha_{i}>0$. Several examples with this setting are considered in for Douglas-Rachford splitting and forward–backward splitting in Sect. 5 and Sect. 6. We know from Theorem 4.2 that the composition is conic if $\alpha_{1}\alpha_{2}<1$. The example in Fig. 8 has $\alpha_{1}=1.7 $ and $\alpha_{2}=0.45$, that satisfies $\alpha_{1}\alpha_{2}=0.76<1$. Theorem 4.2 shows that the composition is of the form $-1.64 {\mathrm{Id}}+ 2.64 N$, where *N* is nonexpansive, i.e., it is 2.64-conic.

**Figure 9:**
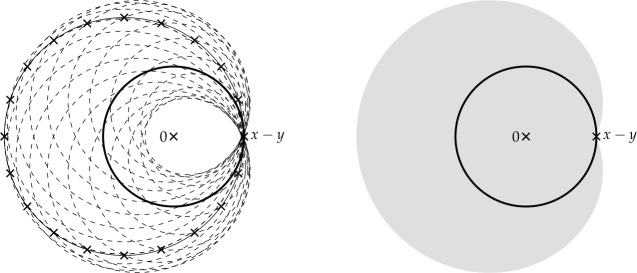
Illustration of composition of $\alpha_{1}$-conic operator and $\alpha_{2}$-averaged operator with $\alpha_{1}=1.7$ and $\alpha_{2}=0.7$

In Example 4.6, we have shown that the assumption $\alpha_{1}\alpha_{2}<1$ is critical for the composition to be conic. Figure 9 illustrates the case $\alpha_{1}=1.7 $ and $\alpha_{2}=0.7 $, which satisfies $\alpha_{1}\alpha_{2}=1.19 >1$, hence Theorem 4.2 cannot be used to deduce that the composition is conic. Indeed, we see from the figure that the composition is not conic. It is impossible to draw a circle that touches the marker at $x-y$ and extends only to the left.

We conclude the conic composed with conic examples with a forward–backward example. The forward–backward splitting operator $J_{\gamma B}({\mathrm{Id}}-\gamma A)$ with *A* $\frac{1}{\beta}$-cocoercive and *B* (maximally) monotone is composed of $\frac{1}{2}$-averaged resolvent $J_{\gamma B}$ and $\frac{\gamma\beta}{2}$-conic forward step $( {\mathrm{Id}}-\gamma A)$. The composition $R=R_{2}R_{1}$ with $R_{i}$ $\alpha_{i}$-conic is conic if $\alpha_{1}\alpha_{2}<1$, Theorem 4.2. In the forward–backward setting, this corresponds to $\gamma\in(0,\frac{4}{\beta})$, which doubles the allowed range compared to guaranteeing an averaged composition. This extended range has been shown before, e.g., in [13, 18].

**Figure 10:**
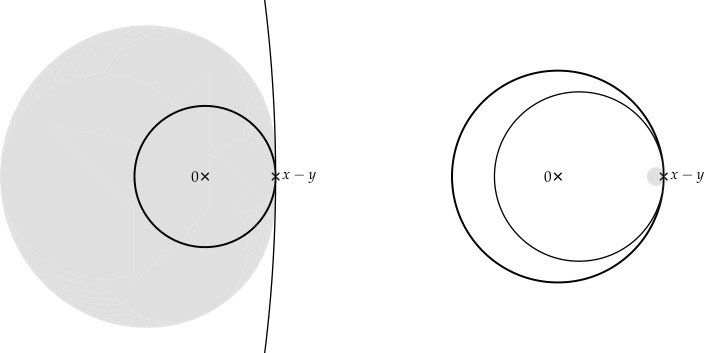
To the left is an illustration of the forward–backward composition $J_{\gamma B}(\mathrm{Id}-\gamma A)$ with $\gamma=\tfrac{3.9}{\beta}$, where $\tfrac{1}{\beta}$ is the cocoercivity constant of *A*. It is a composition between an $\alpha_{1}$-conic operator and an $\alpha_{2}$-averaged operator with $\alpha_{1}=1.95$ and $\alpha_{2}=0.5$. To the right is an illustration of a *θ*-relaxation of the same forward-backward map with $\theta=0.04$

In Fig. 10, we illustrate the forward–backward setting with $\gamma=\frac{3.9}{\beta}$. This corresponds to conic parameters $\alpha_{1}=1.95 $ and $\alpha_{2}=0.5 $, i.e., $R=R_{2}R_{1}$ with $R_{1}=-0.95 {\mathrm{Id}}+1.95 N_{1}$ and $R_{2}=0.5 {\mathrm{Id}}+0.5 N_{2}$. The composition is of the form $-18.99{\mathrm{Id}}+ 19.99 N$, where *N* is nonexpansive, i.e., it is 19.99-conic, Theorem 4.2. The left figure shows the resulting composition and (parts of) the conic approximation. The conic approximation is very large compared to the actual region. This is due to the local behavior around the point $x-y$, where it is almost vertical. As $\gamma\nearrow4\beta$, the exact shape approaches being vertical around $x-y$ and the conic circle approaches to have an infinite radius. For $\gamma>4\beta$, the exact shape extends to the right of $x-y$ (as in the figure above), and the composition will not be conic.

In the right figure, we consider the relaxed forward–backward map $(1-\theta){\mathrm{Id}}+\theta J_{\gamma B}({\mathrm{Id}}-\gamma A)$ with $\theta>0$. If the composition $J_{\gamma B}({\mathrm{Id}}-\gamma A)$ is *α*-conic, it is straightforward to verify that the relaxed map is *θα*-conic. Therefore, any $\theta\in(0,\alpha^{-1})$ gives an *θα*-averaged relaxed forward–backward map. An averaged map is needed to guarantee convergence to a fixed-point when iterated. In the figure, we let $\theta=0.04 $, which satisfies $\theta<\alpha^{-1}\approx0.05$. The approximation is indeed averaged, but the region within which the composition can end up is very small compared to the conic approximation.

#### Scaled averaged and cocoercive compositions

Compositions of scaled averaged and cocoercive operators are also special cases of scaled conic composed with scaled conic operators treated in Theorem 4.2. It covers the forward backward examples in Sect. 6, where identity is shifted between the operators and the sum is (strongly) monotone. The operators in the composition are of the form $R_{1}=\delta_{1}((1-\alpha_{1}){\mathrm{Id}}+\alpha_{1} N_{1})$ and $R_{2}=\frac{\beta_{2}}{2}({\mathrm{Id}}+N_{2})$, where $\alpha_{1}\in(0,1)$, $\delta_{1}>0$, and $\beta_{2}>0$.

**Figure 11:**
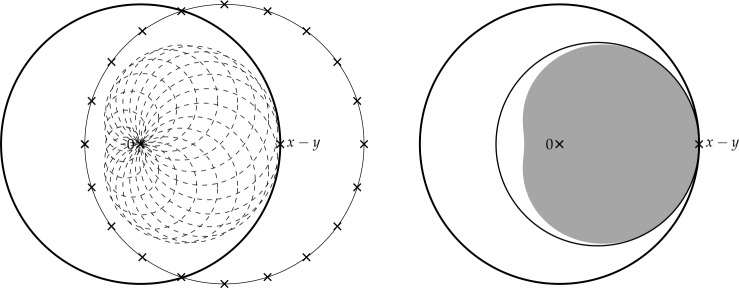
Illustration of composition of 1.6-scaled 0.62-averaged operator with 1.6-cocoercive operator. The composition comes from the forward–backward map $J_{\gamma B}(\mathrm{Id}-\gamma A)$ with $A+0.3\mathrm{Id}$ 1-cocoercive, *B* 0.3-monotone, and $\gamma=2$

In Fig. 11, we consider the forward–backward setting in Theorem 6.5. The forward backward map is $J_{\gamma B}({\mathrm{Id}}-\gamma A)$ and we let $A+0.3 {\mathrm {Id}}$ be 1-cocoercive, *B* be maximally 0.3-monotone. That is, we have shifted 0.3Id from *A* to *B* and the sum is monotone. We use step-length $\gamma=2 $. The proof of Theorem 6.5 shows that, in our setting, $R_{1}$ is 1.6-scaled 0.62-averaged and that $R_{2}$ is 1.6-cocoercive. Theorem 3.4 implies that the composition is of the form $0.27{\mathrm{Id}}+ 0.73 N$, where *N* is nonexpansive, i.e., it is 0.73-averaged.

**Figure 12:**
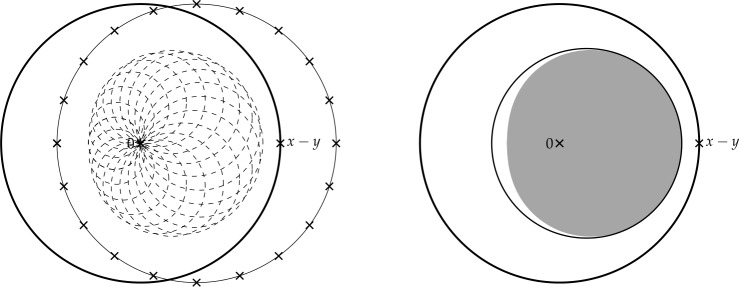
Illustration of composition of 1.4-scaled 0.62-averaged operator with 1.6-cocoercive operator. The composition comes from the forward–backward map $J_{\gamma B}(\mathrm{Id}-\gamma A)$ with $A+0.2\mathrm{Id}$ 1-cocoercive, *B* 0.3-monotone, and $\gamma=2$

Figure 12 considers a similar forward–backward setting, but with a strongly monotone sum. We let $A+0.2 {\mathrm{Id}}$ be 1-cocoercive, *B* be maximally 0.3-monotone, which implies that the sum is 0.1-strongly monotone. We keep step-length $\gamma=2 $. The proof of Theorem 6.5 shows that, in our setting, $R_{1}$ is 1.4-scaled 0.62-averaged and that $R_{2}$ is 1.6-cocoercive. Theorem 3.4 implies that the composition is of the form $0.19{\mathrm{Id}}+ 0.68 N$, where *N* is nonexpansive, i.e., it is 0.87-contractive.

**Figure 13:**
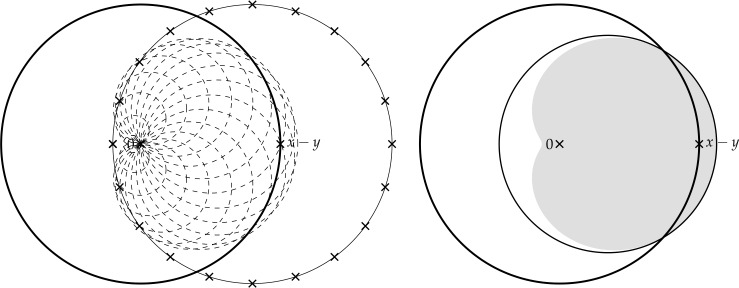
Illustration of composition of 1.8-scaled 0.62-averaged operator with 1.6-cocoercive operator. The composition comes from the forward–backward map $J_{\gamma B}(\mathrm{Id}-\gamma A)$ with $A+0.4\mathrm{Id}$ 1-cocoercive, *B* 0.3-monotone, and $\gamma=2$

The final example in Fig. 13 considers a similar forward–backward setting where the sum is not monotone. We let $A+0.4 {\mathrm{Id}}$ be 1-cocoercive, *B* be maximally 0.3-monotone, which implies that the sum is −0.1-monotone, i.e., it is not monotone. We use step-length $\gamma=2 $. The proof of Theorem 6.5 shows that, in our setting, $R_{1}$ is 1.8-scaled 0.62-averaged and that $R_{2}$ is 1.6-cocoercive. Theorem 3.4 implies that the composition is of the form $0.35{\mathrm{Id}}+ 0.78 N$, where *N* is nonexpansive, i.e., it is 1.12-Lipschitz and not conic, averaged, or contractive.

## Data Availability

Data sharing is not applicable to this article as no datasets were generated or analyzed during the current study.

## Appendix A

### Proof of Lemma 2.3

Indeed, observe that

89 $$ R(\lambda) =(1-2\lambda)\operatorname{Id}+\lambda( \operatorname{Id}+R_{2}R_{1}) $$

and

90 $$ \operatorname{Id}-R(\lambda) =\lambda(\operatorname{Id}-R_{2}R_{1}). $$

In view of (89) and (90) we have

$$\begin{aligned} &\bigl\langle R(\lambda) x-R(\lambda) y \mid\bigl(\operatorname {Id}-R(\lambda)\bigr)x- \bigl( \operatorname {Id}-R(\lambda)\bigr)y \bigr\rangle \\ &\quad =(1-2\lambda)\bigl\langle x-y \mid \operatorname {Id}-R(\lambda))x-\bigl(\operatorname {Id}-R( \lambda)\bigr)y \bigr\rangle \\ &\qquad {} +\lambda^{2}\bigl\langle (x-y)-(R_{2}R_{1}x-R_{2}R_{1}y) \mid (x-y)+(R_{2}R_{1}x-R_{2}R_{1}y) \bigr\rangle \\ &\quad =(1-2\lambda)\bigl\langle x-y \mid \operatorname {Id}-R(\lambda))x-\bigl(\operatorname {Id}-R( \lambda)\bigr)y \bigr\rangle +\lambda^{2} \bigl( \Vert x-y \Vert ^{2}- \Vert R_{2}R_{1}x-R_{2}R_{1}y \Vert ^{2} \bigr) \\ &\quad =(1-2\lambda)\bigl\langle x-y \mid \operatorname {Id}-R(\lambda))x-\bigl(\operatorname {Id}-R( \lambda)\bigr)y \bigr\rangle \\ &\qquad {} +\lambda^{2} \bigl( \Vert x-y \Vert ^{2}- \Vert R_{1}x-R_{1}y \Vert ^{2} + \Vert R_{1}x-R_{1}y \Vert ^{2}- \Vert R_{2}R_{1}x-R_{2}R_{1}y \Vert ^{2} \bigr) \\ &\quad =(1-2\lambda)\bigl\langle x-y \mid\bigl(\operatorname {Id}-R(\lambda)\bigr)x-\bigl( \operatorname {Id}-R( \lambda)\bigr)y \bigr\rangle \\ &\qquad {} +\lambda^{2}\bigl\langle (\operatorname {Id}+R_{1})x-( \operatorname {Id}+R_{1})y \mid( \operatorname {Id}-R_{1})x-( \operatorname {Id}-R_{1})y \bigr\rangle \\ &\qquad {} +\lambda^{2}\bigl\langle (\operatorname {Id}+R_{2})R_{1}x-( \operatorname {Id}+R_{2})R_{1}y \mid(\operatorname {Id}-R_{2})R_{1}x-( \operatorname {Id}-R_{2})R_{1}y \bigr\rangle , \end{aligned}$$

and the conclusion follows. □

## Appendix B

### Proof of Lemma 2.12

(i): Because $\frac{1}{\beta} A$ is nonexpansive, we learn from [2, Example 20.7] that $\operatorname{Id}+\frac{1}{\beta} A$, as is ${\beta}\operatorname{Id}+A$, is maximally monotone. The conclusion now follows in view of e.g., [3, Lemma 2.5]. (ii): This is clear by observing that $\frac{1}{2\beta} ({\beta}\operatorname{Id}+A ) =\frac{1}{2}( \operatorname{Id}+\frac{1}{\beta} A)$. □

## Appendix C

### Proof of Lemma 2.13

Indeed, by assumption, there exist nonexpansive mappings $N_{1}\colon X\to X$ and $N_{2}\colon X\to X$ such that

91 $$ T_{1}=\frac{\beta}{2}\operatorname{Id}+\frac{\beta}{2}N_{1},\qquad T_{2}=\frac{\delta}{2}\operatorname{Id}+\frac{\delta}{2}N_{2}. $$

Now,

92a $$\begin{aligned} \frac{1}{\beta}(T_{1}-T_{2}) &= \frac{1}{\beta} T_{1}- \frac{1}{\beta} T_{2} =\frac{1}{2}\operatorname{Id}+\frac{1}{2}N_{1} - \frac{\delta}{2\beta}\operatorname{Id}-\frac{\delta}{2\beta}N_{2} \end{aligned}$$

92b $$\begin{aligned} &=\frac{\beta-\delta}{2\beta}\operatorname{Id}+\frac{1}{2}N_{1}- \frac{\delta}{2\beta}N_{2}. \end{aligned}$$

Using the triangle inequality, one can directly verify that $\frac{1}{\beta}(T_{1}-T_{2})$ is Lipschitz continuous with a constant $\frac{\beta-\delta}{2\beta}+\frac{1}{2}+\frac{\delta}{2\beta} =1$. The proof is complete. □

## Appendix D

### Proof of Corollary 2.14

(i): It follows from Fact 7.5 that $\mathop{\nabla{f} } _{1}$ (respectively $\mathop{\nabla{f} } _{2}$) is $\frac{1}{\beta}$-cocoercive (respectively $\frac{1}{\delta}$-cocoercive). Now apply Lemma 2.13 with $(T_{1},T_{2})$ replaced by $(\mathop{\nabla{f} } _{1},\mathop{\nabla{f} } _{2})$. (ii): Combine (i) with Fact 7.5 applied with *f* replaced by $f_{1}-f_{2}$. □

## Appendix E

### Proof of Lemma 2.15

(i): Indeed, we have $\delta T=(1-(1-\delta(1-\alpha)))\operatorname{Id}+\delta\alpha N= (1-(1-\delta(1-\alpha)))\operatorname{Id}+(1-\delta(1-\alpha)) \widetilde{N}$, where $\widetilde{N}=((\delta\alpha)/(1-\delta(1-\alpha)))N$. Note that $(\delta\alpha)/(1-\delta(1-\alpha)\le1$, hence *Ñ* is nonexpansive and the conclusion follows. (ii): Clear. □

## Footnotes

1 Let $T\colon X\to X$. Then *T* is *α*-*averaged* if $\alpha\in\, ]0,1 [$ and nonexpansive $N\colon X\to X$ exists such that $T=(1-\alpha)\operatorname {Id}+\alpha N$.

2 Let $T\colon X\to X$. Then *T* is *α*-*conically nonexpansive* if $\alpha\in\, ]0,\infty [$ and nonexpansive $N\colon X\to X$ exists such that $T=(1-\alpha)\operatorname {Id}+\alpha N$.

3 Let $T\colon X\to X$, and let $\beta>0$. Then *T* is $\frac{1}{\beta}$-*cocoercive* if nonexpansive $N:X\to X$ exists such that $T=\frac{\beta}{2}({\mathrm{Id}}+N)$.

4 The paper [1] appeared online while putting the finishing touches on this paper. Partial results of this work were presented by the second author at the *Numerical Algorithms in Nonsmooth Optimization* workshop at Erwin Schrödinger International Institute for Mathematics and Physics (ESI) in Vienna in February 2019 and at the *Operator Splitting Methods in Data Analysis* workshop at the Flatiron Institute, in New York in March 2019. Both workshops predate [1].

5 Let $A\colon XX$ be an operator. The *resolvent* of *A*, denoted by $J_{A}$, is defined by $J_{A}=(\operatorname {Id}+A)^{-1}$, and the *reflected resolvent* of *A*, denoted by $R_{A}$, is defined by $R_{A}=2J_{A}-\operatorname {Id}$

6 In passing, we mention that [2, Proposition 26.1(iv)] assume that *A* and *B* are maximally monotone, which is not required here. However, the proof is the same.

7 Here and elsewhere, we use ${\mathbb {R}}_{+}$ to denote the interval $[0,+\infty [$.

8 The assumption that $\beta\in{\mathbb {R}}_{+} $ is not restrictive. Indeed, since *N* is nonexpansive, an operator admits an $(\alpha,\beta)$-I-N decomposition if and only if it admits an $(\alpha,-\beta)$-I-N decomposition. This is the reason why we define it only for nonnegative *β*.

9 Let *C* be a nonempty, closed convex subset of *X*. Here and elsewhere, we shall use $N_{C}$ to denote the *normal cone operator* associated with *C*, defined by $N_{C}(x)=\{ {u\in X} \mid{\sup\langle C-x \mid u \rangle\le0} \}$ if $x\in C$; and $N_{C}(x)=\varnothing$, otherwise.
